# FCGR2A defines prognostic immune subtypes and drives tumor progression in hepatocellular carcinoma

**DOI:** 10.3389/fimmu.2025.1641420

**Published:** 2025-10-24

**Authors:** Deyuan Zhong, Yuxin Liang, Hongtao Yan, Xing Chen, YaHui Chen, Shuoshuo Ma, Yuhao Su, Fei Wang, Xinpei Chen, Qinyan Yang, Zhengwei Leng, Ming Wang, Xiaolun Huang

**Affiliations:** ^1^ Department of Liver Transplantation Center and Hepato-Biliary-Pancreatic (HBP) Surgery, Sichuan Clinical Research Center for Cancer, Sichuan Cancer Hospital & Institute, Sichuan Cancer Center, School of Medicine, University of Electronic Science and Technology of China, Chengdu, China; ^2^ State Key Laboratory of Quality Research in Chinese Medicine, Macau Institute for Applied Research in Medicine and Health, Macau University of Science and Technology, Macao, Macao SAR, China; ^3^ Center for Natural Products Research, Chengdu Institute of Biology, Chinese Academy of Sciences, Chengdu, China

**Keywords:** hepatocellular carcinoma, FCGR2A, immune subtypes, immunotherapy, bioinformatics, functional validation

## Abstract

**Background:**

The immunosuppressive nature of the HCC tumor microenvironment limits the effectiveness of current immunotherapeutic strategies. Identifying key immune-related regulators is essential for improving patient stratification and therapeutic outcomes.

**Methods:**

Transcriptomic data from TCGA and GEO datasets were integrated to screen IRDEGs. Functional enrichment, co-expression, and PPI network analyses were performed to explore the biological context. Consensus clustering based on hub gene expression was used to define immune-related molecular subtypes. Immune infiltration characteristics, immune checkpoint expression, TIDE and IPS scores, and predicted immunotherapy responses were compared. FCGR2A expression was validated in clinical HCC tissues by immunohistochemistry and western blotting. *In vitro* assays evaluated the effects of FCGR2A knockdown on HCC cell proliferation, migration, and invasion.

**Results:**

A total of 21 IRDEGs were identified, among which FCGR2A was consistently upregulated and associated with poor prognosis. Enrichment analysis indicated significant involvement in immune activation and inflammatory signaling pathways. PPI network analysis identified nine hub genes, including FCGR2A. Consensus clustering revealed two distinct immune-related molecular subtypes with marked differences in immune infiltration patterns, immune checkpoint profiles, TIDE and IPS scores. GSEA demonstrated subtype-specific activation of antigen processing, T cell signaling, and inflammatory pathways. Experimental validation confirmed elevated FCGR2A expression in HCC tissues. Functional assays showed that FCGR2A knockdown significantly inhibited HCC cell proliferation, migration, and invasion.

**Conclusions:**

FCGR2A acts as both a prognostic biomarker and an immune regulatory hub in HCC, anchoring a broader gene network that defines immune subtypes and predicts therapeutic responsiveness. Incorporating FCGR2A-based stratification may optimize immunotherapeutic strategies for HCC.

## Introduction

HCC is the predominant subtype of primary liver cancer, accounting for approximately 90% of global liver cancer cases, and consistently ranks among the leading causes of cancer-related morbidity and mortality worldwide (1). Due to the insidious onset and lack of highly sensitive diagnostic biomarkers, most HCC patients are diagnosed at an advanced stage, at which point conventional therapies offer limited efficacy. The substantial intratumor heterogeneity and drug resistance further complicate clinical management (2). Thus, HCC treatment remains a formidable challenge, underscoring the urgent need for novel therapeutic strategies.

In recent years, immunotherapies such as ICIs have demonstrated clinical benefits in advanced HCC, with several pivotal phase III trials confirming survival advantages (3–6). Nevertheless, the application of ICIs in HCC faces multiple obstacles. Not all patients respond to immunotherapy, and the immunosuppressive microenvironment, together with the intrinsic heterogeneity of HCC, contributes to primary or acquired resistance in a significant proportion of cases (7). Currently, the absence of reliable biomarkers for predicting therapeutic responsiveness hinders optimal clinical decision-making (8, 9).

Given this background, the identification of novel therapeutic targets and prognostic biomarkers is crucial to improving outcomes in HCC (10). In-depth exploration of the tumor immune microenvironment and discovery of key immune-related molecules may overcome treatment resistance and enhance immunotherapeutic efficacy (11). This study focuses on the characterization of IRDEGs in HCC, which are intimately involved in antitumor immune responses and may contribute to tumor progression and immune evasion when dysregulated. Prior studies across various cancers have demonstrated that profiling IRDEGs can unveil novel therapeutic targets for personalized treatment and improve clinical efficacy (12, 13). By integrating multi-omics analyses with experimental validation, this study aims to systematically identify immune-regulatory IRDEGs in HCC, uncover their mechanistic roles, and propose novel immune biomarkers and therapeutic candidates, providing a theoretical and technical foundation for precision immunotherapy in HCC.

## Materials and methods

### Data acquisition and preprocessing

RNA-seq data and clinical information for 368 HCC tumor samples and 50 adjacent normal tissues were obtained from the TCGA-LIHC dataset via the R package *TCGAbiolinks (* 14). Raw count data were normalized to FPKM format, and clinical annotations were retrieved from the UCSC Xena platform (15). Two microarray datasets, GSE10143 (16) (GPL5474, 80 HCC samples) and GSE14520 (17) (GPL3921, 225 HCC and 220 normal liver samples), were downloaded from the GEO database using the R package *GEOquery (* 18, 19) (**Table 1**). A total of 124 IRGs were retrieved from the GeneCards (20, 21) using the keyword “Immunotherapy” and filtered by criteria: protein-coding and relevance score >3. (**Supplementary Table S1**). Batch effects across GEO datasets were corrected using the sva package (22), and expression matrices were normalized using limma (23). Principal Component Analysis (24) was used to assess batch correction.

**Table 1:** Baseline table with LIHC patients characteristics.

Characteristics	Overall
Age, median (IQR)	62 (52, 69.75)
Gender, n (%)	
Male	277 (66.3%)
female	141 (33.7%)
Stage, n (%)	
Stage I	190 (49.2%)
Stage II	96 (24.9%)
Stage III&IV	100 (25.9%)

To provide an integrated perspective on the analytical strategy employed in this study, a comprehensive schematic diagram is presented in Flow Chart for the Comprehensive Analysis of FCGR2A. This workflow outlines the sequential steps from multi-dataset integration and immune-related gene screening to FCGR2A-centric analyses, co-expression network construction, consensus clustering, and evaluation of immunotherapy responsiveness.

Flow Chart for the Comprehensive Analysis of FCGR2A.

### Identification of differentially expressed genes

DEGs were identified between tumor and normal samples in the TCGA-LIHC dataset using DESeq2 (|logFC| > 0.25, p < 0.05) (25). For GEO datasets, DEGs were identified using limma with the same thresholds. A volcano plot was generated using ggplot2 (v3.4.4). Volcano plots and heatmaps were generated using ggplot2 and pheatmap (v1.0.12), respectively, to visualize expression differences of IRDEGs.

### Functional enrichment analysis

GO (26), KEGG (27) and GSEA (28) were conducted using the clusterProfiler package (v4.10.0) (29). GO and KEGG analyses were based on IRDEGs, while GSEA was performed on the full gene expression matrix ranked by logFC using the MSigDB c2 gene set (v2023.2.Hs). Parameters included seed = 2020, gene set size = 10–500, adjusted p < 0.05, and FDR < 0.25.

### Prognostic model construction

Patients were stratified into high- and low-expression groups based on the optimal cut-off value of FCGR2A. Kaplan–Meier (30) survival curves and time-dependent ROC (31) curves were generated using survival and survivalROC packages. Cox regression was used to determine independent prognostic value, with visualizations via forest plots.

### PPI network and hub gene identification

A PPI network of FCGR2A co-expressed genes was constructed using STRING (score > 0.400) (32). Hub genes were identified using CytoHubba (algorithms: MCC, Degree, MNC, EPC, Closeness) (33), and the top 10 genes from each method were intersected. Venn diagrams visualized the overlapping hub genes.

### Multilayer regulatory networks

Regulatory relationships were retrieved from multiple databases: TF–mRNA from ChIPBase (34), miRNA–mRNA from TarBase (http://www.microrna.gr/tarbase), RBP–mRNA from StarBase (https://starbase.sysu.edu.cn/), and drug–gene interactions from CTD (https://ctdbase.org/). All networks were visualized using Cytoscape (35).

### Immune infiltration and molecular subtyping

ssGSEA (36) was applied using the GSVA package to estimate immune cell infiltration levels. Spearman correlation and heatmaps were used to evaluate immune cell relationships. Hub gene–immune cell associations were visualized using bubble plots. Consensus clustering (37) based on hub gene expression was performed using ConsensusClusterPlus (38) to identify HCC subtypes. CDF curves and consensus matrices determined the optimal number of clusters. Heatmaps and group comparison plots showed hub gene differences across subtypes.

### Immunotherapy response prediction

Expression differences of 47 ICGs across subtypes were assessed using the Mann–Whitney U test (39) (**Supplementary Table S2**). TIDE scores were downloaded from the TIDE platform to predict immune evasion and therapy response (40, 41) (http://tide.dfci.harvard.edu). IPS from the TCIA database quantified tumor immunogenicity and were compared between subtypes (42) (https://tcia.at/home). TMB (43) (https://www.cbioportal.org/) and MSI data from cBioPortal were also analyzed between groups.

### Somatic mutation and pathway analysis

Masked somatic mutation data were obtained from TCGA and processed with VarScan. Mutation profiles were analyzed using maftools (44) (v2.10.0). Differences between FCGR2A high- and low-expression groups were visualized using ggplot2. Oncogenic pathway enrichment was analyzed using maftools functions OncogenicPathways and PlotOncogenicPathways.

### Patient tissue specimens and immunohistochemistry

Formalin-fixed, paraffin-embedded HCC tissues and paired adjacent non-tumorous liver tissues were obtained from Sichuan Cancer Hospital, Chengdu, China. None of the patients had received preoperative chemotherapy, radiotherapy, or immunotherapy prior to surgical resection. Clinicopathological information was collected from medical records, and all tissue samples were histopathologically confirmed by two independent pathologists.

FFPE tissue specimens from HCC and matched adjacent non-tumor tissues were sectioned at 4 μm thickness. After deparaffinization and rehydration, antigen retrieval was performed using 10 mM sodium citrate buffer (pH 6.0) in a pressure cooker for 10 minutes. Endogenous peroxidase activity was quenched with 3% hydrogen peroxide for 10 minutes, followed by blocking with 5% BSA for 30 minutes at room temperature. Sections were incubated overnight at 4 °C with a rabbit anti-FCGR2A primary antibody (Abcam, ab134045, dilution 1:200), followed by incubation with HRP-conjugated secondary antibody (ZSGB-BIO, China, PV-9001) for 30 minutes at 37 °C. Signal was developed using DAB substrate kit (ZSGB-BIO), and nuclei were counterstained with hematoxylin. Stained sections were scanned using a PANNORAMIC 250 digital slide scanner (3DHISTECH). Quantitative analysis of FCGR2A expression was performed using HALO software (v3.3; Indica Labs). Positive staining was defined as brown cytoplasmic or membranous signal. Expression scores were calculated based on H-score method: H-score = (% weak ×1) + (% moderate ×2) + (% strong ×3), yielding a total score range of 0–300.

### Cell culture and *in vitro* experiments

THLE-2 normal liver cells (CL-0833, Procell) and human HCC cell lines SNU-878 (h458, iCell), Huh-7 (h080, iCell), and HepG2 (h092, iCell) were cultured in RPMI-1640 or DMEM (Gibco, USA) supplemented with 10% fetal bovine serum (FBS, Gibco) and 1% penicillin/streptomycin at 37 °C in a humidified incubator with 5% CO_2_. For gene silencing assays, SNU-878 cells were seeded in 6-well plates at a density of 2 × 10^5^ cells/well. Transfection was conducted using the Hieff Trans™ Liposomal Transfection Reagent (YEASEN, Shanghai, China) according to the manufacturer’s instructions. Briefly, 75pmol of siRNA was mixed with 42.5μL transfection buffer and 7.5μL Plus reagent, incubated for 10 min to form a siRNA-lipid complex, and added to cells after 6 h of attachment. Medium was replaced after 6 h with fresh complete medium. Four siRNAs targeting FCGR2A were synthesized by GenePharma (Shanghai, China) with the following sequences: FCGR2A-Homo-12: Sense: 5′-AUGACUAUGAGACCCAAATT-3′ and Antisense: 5′-UUUGGGUCUCAUAGUCAUTT-3′; FCGR2A-Homo-137: Sense: 5′-GAAACUUGAGCCCCCGUGGTT-3′ and Antisense: 5′-CCACGGGGGCUCAAGUUUCTT-3′; FCGR2A-Homo-507: Sense: 5′-CAUUUGGAUUCCACCUUCUUTT-3′ and Antisense: 5′-AGAAGGUGGAAUCCAAAUGTT-3′; FCGR2A-Homo-678 Sense: 5′-AUUUGGCACUGCUGUAGCAGTT-3′ and Antisense: 5′-CUGCUACAGCAGUGCCAAUTT-3′. Knockdown efficiency was evaluated by Western blotting 48 hours post-transfection. The most effective siRNA sequence was used for subsequent functional assays.

### Western blotting

Total protein was extracted from transfected and control cells using RIPA lysis buffer (Beyotime, China) supplemented with protease and phosphatase inhibitors. Protein concentrations were determined using a BCA Protein Assay Kit (Thermo Fisher Scientific, USA). Equal amounts of protein (30μg per sample) were separated by SDS-PAGE on 10% polyacrylamide gels, and then transferred onto PVDF membranes (Millipore, USA). Membranes were blocked with 5% non-fat milk in TBST for 1 hour at room temperature and incubated overnight at 4 °C with primary antibodies against FCGR2A (Abcam, ab182958, 1:1000) and β-actin (Cell Signaling Technology, #4970, 1:5000). After washing, membranes were incubated with appropriate HRP-conjugated secondary antibodies (1:5000, CST) for 1 hour at room temperature. Protein bands were visualized using enhanced chemiluminescence (ECL, Thermo Scientific) and imaged with a ChemiDoc™ XRS+ System (Bio-Rad, USA). Band intensities were quantified using ImageJ software (v2.3.0, NIH, USA) and normalized to β-actin.

### Cell functional assays

Cell viability was assessed using the Cell Counting Kit-8 (CCK-8, Dojindo, Japan) according to the manufacturer’s instructions. Transfected and control SNU-878 cells were seeded into 96-well plates (5 × 10³ cells/well) and incubated for 24, 48, and 72 hours. At each time point, 10μL of CCK-8 reagent was added per well and incubated for 2 hours. Absorbance was measured at 450 nm using a microplate reader (BioTek, USA).

Wound healing assays were conducted to evaluate cell migratory capacity. Transfected SNU-878 cells were seeded into 6-well plates and grown to ~90% confluency. A straight scratch was made using a 200μL pipette tip, and detached cells were gently removed with PBS. Cells were then cultured in serum-free medium and imaged at 0 and 24 hours under an inverted microscope (Olympus, Japan). Migration rate was calculated by measuring the wound area using ImageJ software.

Invasion assays were performed using Transwell chambers (8μm pore size, Corning, USA) pre-coated with Matrigel (BD Biosciences, USA). A total of 1 × 10^5^ transfected cells in serum-free medium were seeded into the upper chamber, while the lower chamber was filled with medium containing 10% FBS as chemoattractant. After 24 hours of incubation at 37 °C, non-invading cells were removed, and invaded cells on the lower surface were fixed with 4% paraformaldehyde, stained with 0.1% crystal violet, and counted under a microscope.

### Statistical analysis

All analyses were conducted using R (v4.3.0) and SPSS 27.0. Student’s t-test or Mann–Whitney U test was applied as appropriate. The Kruskal–Wallis test was used for comparisons among multiple groups. Spearman correlation assessed variable relationships. A p-value < 0.05 was considered statistically significant. All *in vitro* experiments, including CCK-8 assays, wound healing, and Transwell assays, were independently performed at least three times. Data are presented as mean ± SD from three biological replicates unless otherwise specified.

## Results

### Identification of IDREGs and initial screening of FCGR2A

Firstly, to identify immune-related gene signatures in HCC, we first performed differential expression analyses using the liver cancer expression datasets GSE10143 and GSE14520 (**Table 2**). After batch effect correction and normalization using the sva package, PCA and boxplot visualizations confirmed effective harmonization across the merged datasets (**Supplementary Figures S1A–D**).

**Table 2:** GEO microarray chip information.

	GSE10143	GSE14520
Platform	GPL5474	GPL3921
Species	Homo sapiens	Homo sapiens
Tissue	Liver	Liver
Samples in LIHC group	80	225
Samples in Control group	/	220
Reference	PMID: 31344396	PMID: 39718737

**Figure 1:**
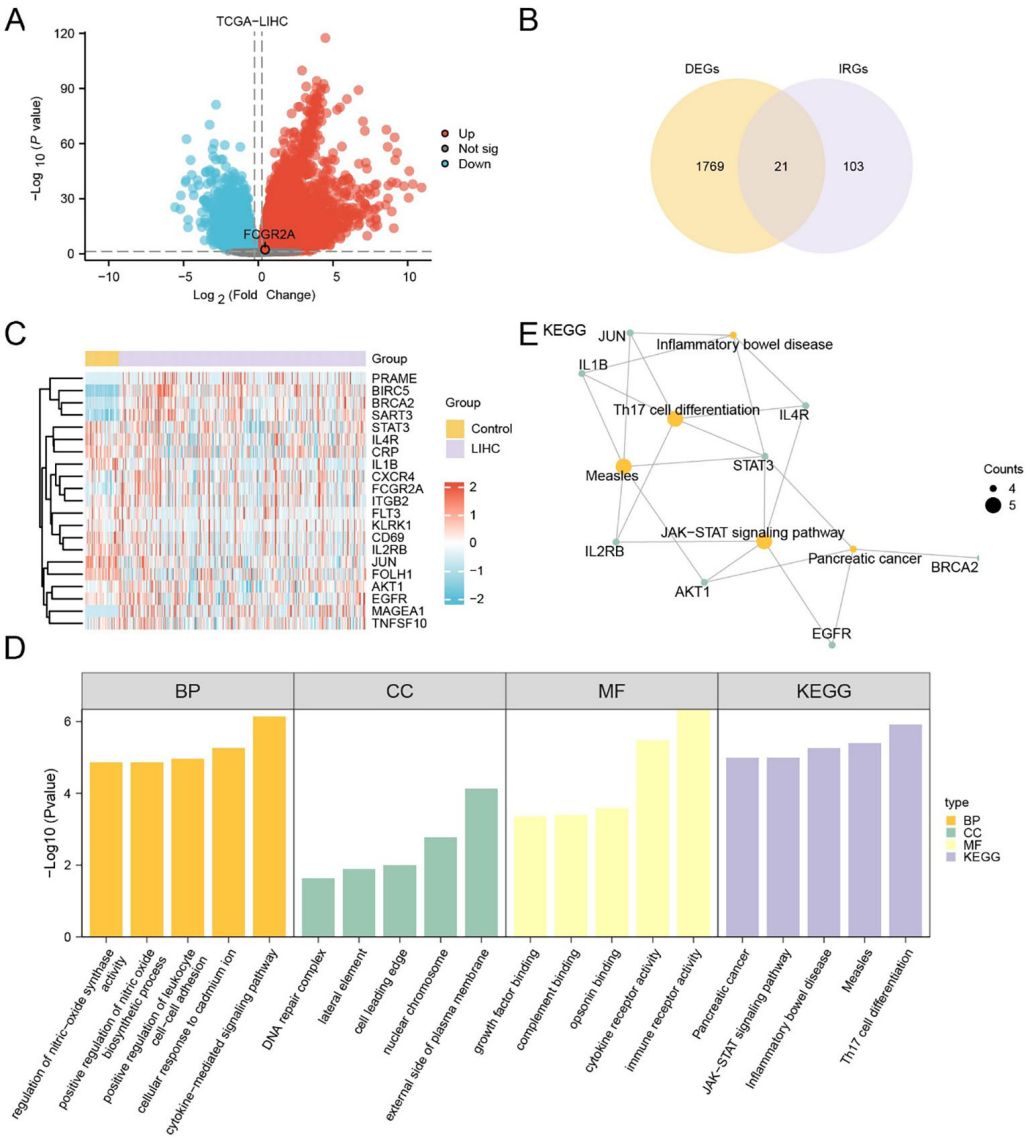
Identification and functional characterization of IRDEGs in HCC. **(A)** Volcano plot showing DEGs in the TCGA-LIHC cohort. Red and blue dots represent significantly upregulated and downregulated genes, respectively (|log_2_FC| > 1.5, *adj. p* < 0.05); gray dots indicate non-significant genes. **(B)** Venn diagram showing the overlap between DEGs and known IRGs, identifying 21 IRDEGs for further analysis. **(C)** Heatmap displaying the expression profiles of the 21 IRDEGs in TCGA-LIHC tumor versus control samples. Red and blue indicate higher and lower expression levels, respectively. **(D)** Barplot summarizing GO and KEGG enrichment analysis results of the IRDEGs, including top terms from BP, CC, MF, and KEGG pathways. **(E)** Gene-concept network plot of significantly enriched KEGG pathways based on the 21 IRDEGs. Node size reflects the gene count per term.

In the TCGA cohort, 12,596 DEGs were identified (|log_2_FC| > 0.25, p < 0.05), with 7,853 upregulated and 4,743 downregulated (**Figure 1A**). Similarly, 2,630 DEGs (1,467 up, 1,163 down) were obtained from the merged GEO datasets (**Supplementary Figure S3A**). Venn diagram analysis revealed 1,790 overlapping DEGs between TCGA and GEO cohorts (**Supplementary Figures S3B–C**), which were intersected with a predefined IRG set to yield 21 IRDEGs (**Figure 1B**; **Supplementary Table S3**). These included: BIRC5, MAGEA1, PRAME, BRCA2, SART3, FCGR2A, JUN, STAT3, IL1B, IL4R, FOLH1, CD69, AKT1, IL2RB, FLT3, EGFR, CRP, TNFSF10, KLRK1, CXCR4, and ITGB2.

Expression heatmaps in both TCGA (**Figure 1C**) and GEO (**Supplementary Figure S3D**) datasets confirmed consistent differential expression patterns of the 21 IRDEGs between HCC and normal tissues. Further statistical comparison validated that most IRDEGs showed significant differential expression across both datasets, including FCGR2A (**Supplementary Figures S2A, B**).

Functional enrichment analyses demonstrated that IRDEGs were significantly involved in immune- and inflammation-related pathways. GO analysis revealed enrichment in cytokine-mediated signaling, leukocyte adhesion, and nitric oxide biosynthesis (**Figure 1D**; **Table 3**). Molecular functions such as opsonin binding, immune receptor activity, and complement binding were also prominent. KEGG analysis highlighted pathways including Th17 cell differentiation, JAK–STAT signaling, and pancreatic cancer. The enrichment network (**Figure 1E**) and complementary GO-term networks (**Supplementary Figures S3E–G**) further illustrated the functional landscape of IRDEGs and positioned FCGR2A as a hub gene involved in multiple immune-regulatory processes.

**Table 3:** Result of GO and KEGG enrichment analysis for DEGs.

Ontology	ID	Description	Gene ratio	Bg ratio	Pvalue	P.adjust	Qvalue
BP	GO:0019221	cytokine-mediated signaling pathway	7/21	492/18614	7.28E-07	1.14E-03	5.71E-04
BP	GO:0071276	cellular response to cadmium ion	3/21	31/18614	5.45E-06	4.15E-03	2.08E-03
BP	GO:1903039	positive regulation of leukocyte cell-cell adhesion	5/21	273/18614	1.10E-05	4.15E-03	2.08E-03
BP	GO:0045429	positive regulation of nitric oxide biosynthetic process	3/21	42/18614	1.38E-05	4.15E-03	2.08E-03
BP	GO:0050999	regulation of nitric-oxide synthase activity	3/21	42/18614	1.38E-05	4.15E-03	2.08E-03
CC	GO:0009897	external side of plasma membrane	5/21	426/19518	7.38E-05	5.38E-03	4.27E-03
CC	GO:0000228	nuclear chromosome	3/21	223/19518	1.68E-03	6.14E-02	4.87E-02
CC	GO:0031252	cell leading edge	3/21	422/19518	9.99E-03	1.90E-01	1.51E-01
CC	GO:0000800	lateral element	1/21	12/19518	1.28E-02	1.90E-01	1.51E-01
CC	GO:1990391	DNA repair complex	1/21	22/19518	2.34E-02	1.90E-01	1.51E-01
MF	GO:0140375	immune receptor activity	5/21	141/18369	4.57E-07	6.04E-05	3.32E-05
MF	GO:0004896	cytokine receptor activity	4/21	92/18369	3.30E-06	2.18E-04	1.20E-04
MF	GO:0001846	opsonin binding	2/21	21/18369	2.58E-04	1.14E-02	6.25E-03
MF	GO:0001848	complement binding	2/21	26/18369	3.98E-04	1.16E-02	6.37E-03
MF	GO:0019838	growth factor binding	3/21	132/18369	4.39E-04	1.16E-02	6.37E-03
KEGG	hsa04659	Th17 cell differentiation	5/16	109/8541	1.21E-06	1.98E-04	8.25E-05
KEGG	hsa05162	Measles	5/16	139/8541	4.02E-06	3.02E-04	1.26E-04
KEGG	hsa05321	Inflammatory bowel disease	4/16	66/8541	5.52E-06	3.02E-04	1.26E-04
KEGG	hsa04630	JAK-STAT signaling pathway	5/16	168/8541	1.02E-05	3.10E-04	1.29E-04
KEGG	hsa05212	Pancreatic cancer	4/16	77/8541	1.02E-05	3.10E-04	1.29E-04

### Prognostic and functional implications of FCGR2A in HCC

**Figure 2:**
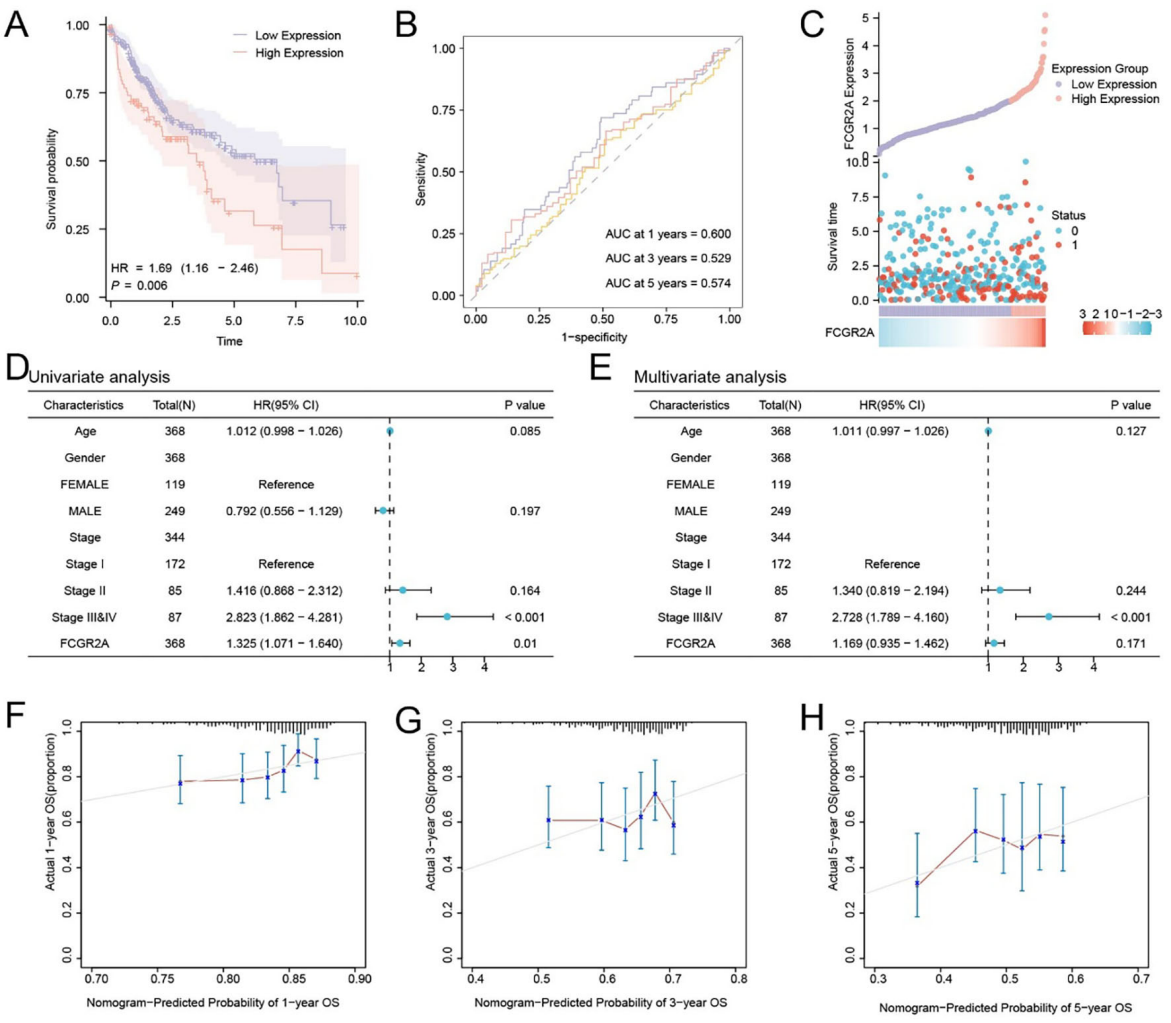
Prognostic significance and survival prediction value of FCGR2A in hepatocellular carcinoma. **(A)** Kaplan–Meier survival analysis of FCGR2A in the TCGA-LIHC cohort. **(B)** Time-dependent ROC curves of FCGR2A expression for predicting 1-, 3-, and 5-year overall survival. **(C)** Distribution of FCGR2A expression, survival time, and survival status in HCC patients. **(D, E)** Univariate and multivariate Cox regression analyses of clinical variables and FCGR2A expression in relation to overall survival. **(F–H)** Calibration plots for a nomogram model integrating FCGR2A expression to predict 1-, 3-, and 5-year overall survival probabilities in HCC patients.

To assess the prognostic significance of FCGR2A in LIHC, patients from the TCGA-LIHC cohort were stratified into high- and low-expression groups based on the optimal cutoff value. Kaplan–Meier analysis revealed significantly poorer OS in the high-expression group (p < 0.05; **Figure 2A**). Time-dependent ROC curves indicated moderate predictive accuracy, with AUCs ranging from 0.5 to 0.7 at 1-, 3-, and 5-year time points (**Figure 2B**). A risk distribution plot demonstrated increased mortality in patients with elevated FCGR2A expression (**Figure 2C**).

Univariate Cox regression analysis identified FCGR2A and clinical stage as potential prognostic variables (**Figure 2D**). Multivariate analysis further confirmed FCGR2A as an independent prognostic factor for OS (**Figure 2E**). Calibration curves showed good concordance between predicted and actual survival probabilities, especially at 1 year (**Figures 2F–H**), supporting the model’s predictive validity.

To explore the functional relevance of FCGR2A, we constructed a PPI network using the GeneMANIA database, which revealed extensive connections between FCGR2A and immune-regulatory genes (**Supplementary Figure S4A**; **Supplementary Table S4**). Additionally, multilayered regulatory networks were established, including transcription factors (**Supplementary Figure S4B**; **Supplementary Table S5**), miRNAs (**Supplementary Figure S4C**; **Supplementary Table S6**), RNA-binding proteins (**Supplementary Figure S4D**; **Supplementary Table S7**), and candidate compounds (**Supplementary Figure S4E**; **Supplementary Table S8**). These findings suggest that FCGR2A may play a multifaceted role in tumor biology and immune regulation, highlighting its potential as both a prognostic biomarker and a therapeutic target in LIHC.

### Experimental validation of FCGR2A as a functional promoter in HCC

**Figure 3:**
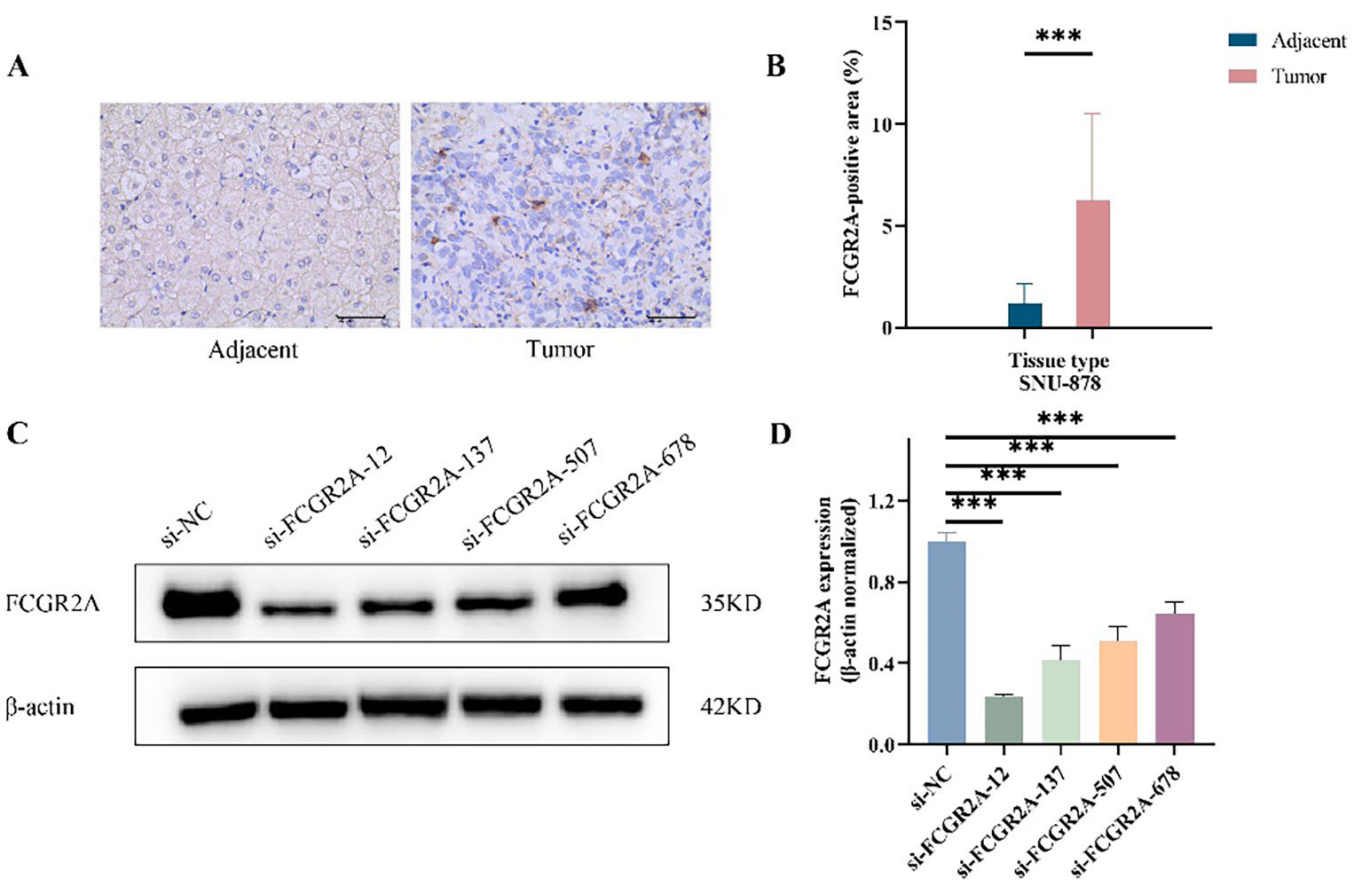
FCGR2A protein expression in HCC tissues and liver cell lines. **(A)** Representative IHC staining of FCGR2A in HCC tissues and matched adjacent non-tumor tissues. **(B)** Quantitative scoring of IHC staining for FCGR2A expression in tumor and adjacent tissues. **(C)** Western blot analysis showing the knockdown efficiency of four siRNA constructs targeting FCGR2A in SNU-878 cells. **(D)** Densitometric quantification of FCGR2A protein levels normalized to β-actin after siRNA transfection. ***p < 0.001.

To validate the tumor-specific expression of FCGR2A at the protein level, we performed IHC on paired HCC and adjacent tissues. FCGR2A was predominantly localized to the membrane and cytoplasm, showing significantly higher expression in tumor tissues (p < 0.001; **Figures 3A,B**). Western blot (WB) analysis further confirmed this upregulation, with tumor tissues displaying a 5-fold increase in FCGR2A levels compared to adjacent liver (6.24 ± 4.28% vs. 1.20 ± 0.97%).

To identify suitable *in vitro* models, FCGR2A protein expression was evaluated in normal hepatocytes (THLE-2) and HCC cell lines (HepG2, Huh-7, and SNU-878). All cancer cell lines exhibited elevated FCGR2A expression, with the highest level observed in SNU-878 cells (p < 0.01), which were therefore selected for subsequent functional assays (**Supplementary Figure S8**). The knockdown efficiency of four siRNA constructs targeting FCGR2A in SNU-878 cells was further confirmed by Western blotting (**Figures 3C, D**).

**Figure 4:**
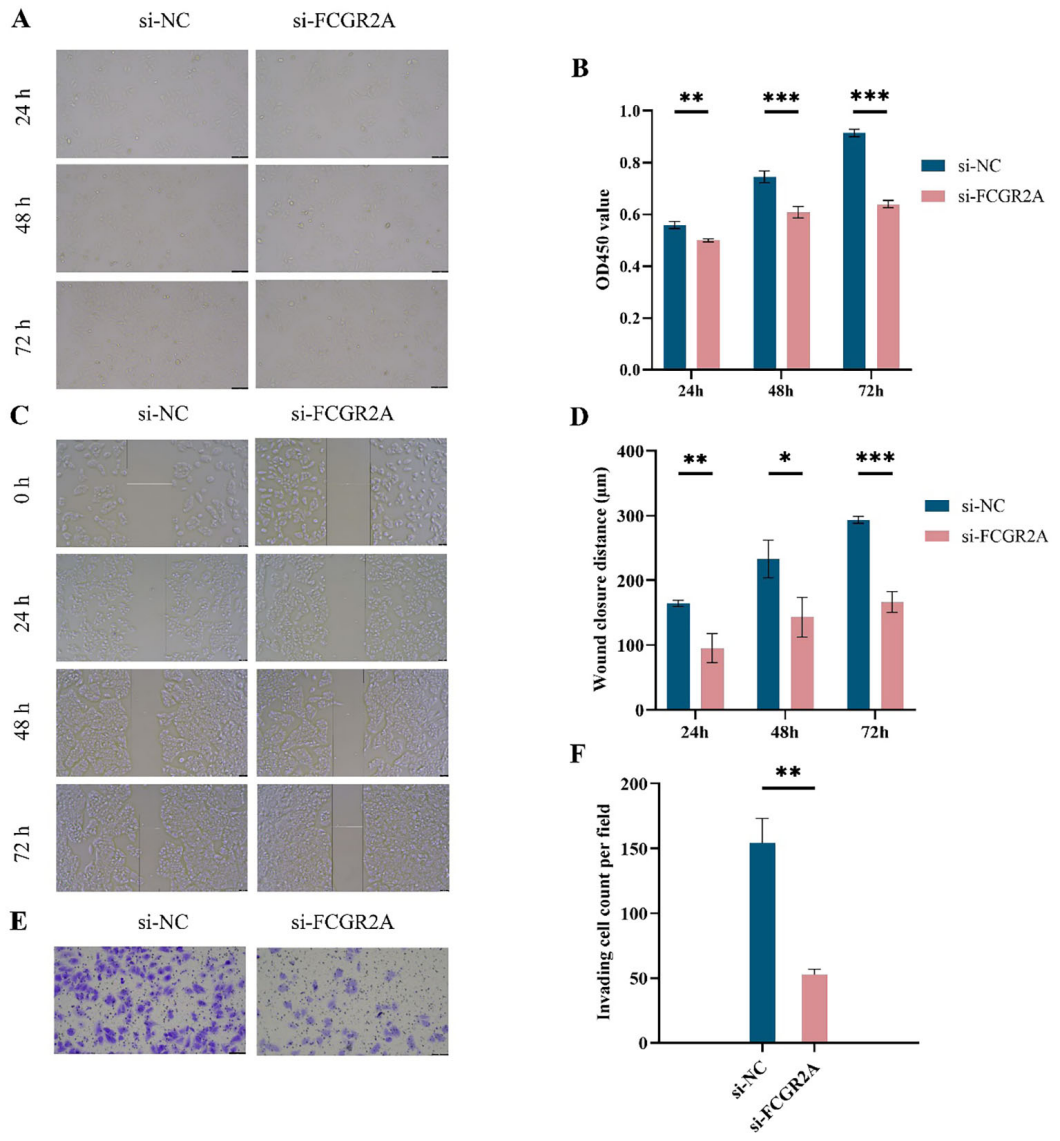
Functional assays of FCGR2A knockdown in liver cancer cells. **(A)** CCK-8 assay images of SNU-878 cells transfected with si-NC or si-FCGR2A at 24 h, 48 h, and 72 h **(B)** Quantification of OD450 values from the CCK-8 assay at different time points. **(C)** Representative wound healing images at 0 h, 24 h, 48 h, and 72 h post-scratch in si-NC and si-FCGR2A groups. **(D)** Quantitative analysis of wound closure distance. **(E)** Representative images from Transwell invasion assay showing cell invasion capacity. **(F)** Quantification of the number of invading cells per field. * p < 0.05; ** p < 0.01; *** p < 0.001.

After selecting SNU-878 as the cell model with the highest FCGR2A expression, functional validation was performed using siRNA-mediated FCGR2A knockdown. CCK-8 assays showed that silencing FCGR2A significantly suppressed cell proliferation at 24, 48, and 72 hours (**Figures 4A, B**). Wound healing assays demonstrated reduced migratory ability following FCGR2A inhibition (**Figures 4C,D**), while Transwell assays revealed markedly impaired invasion capacity (**Figures 4E, F**). These findings collectively support a pro-tumorigenic role of FCGR2A in HCC progression.

### Functional enrichment and co-expression network analysis of FCGR2A

**Figure 5:**
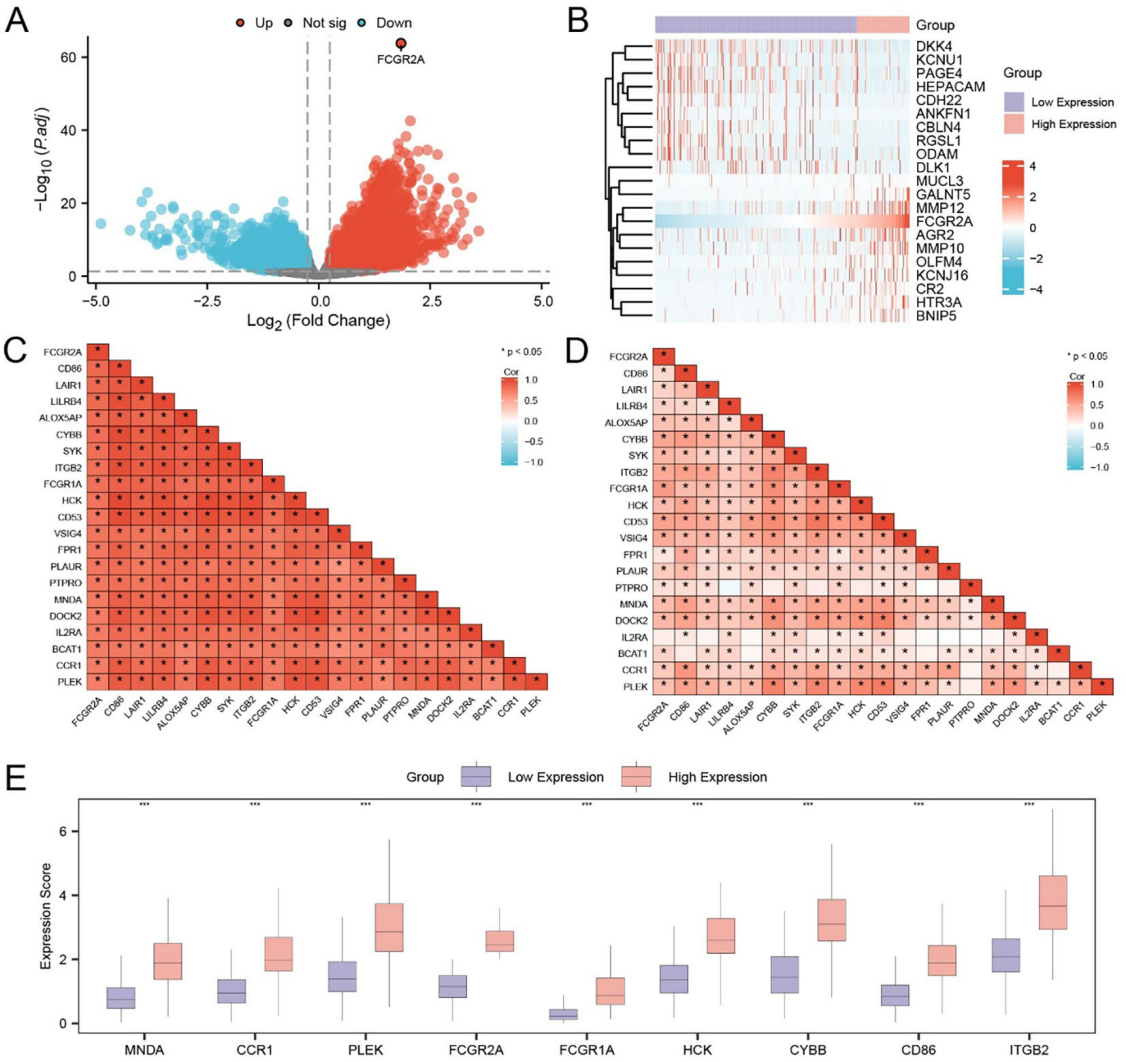
Identification and expression pattern of FCGR2A-related hub genes in HCC. **(A)** Volcano plot showing DEGs between high and low FCGR2A expression groups in the TCGA-LIHC cohort. **(B)** Heatmap of the top 20 DEGs associated with FCGR2A expression. **(C, D)** Pearson correlation matrices of FCGR2A-related genes in the low **(C)** and high **(D)** FCGR2A expression groups, respectively. **(E)** Boxplots showing the expression levels of the 9 hub genes (MNDA, CCR1, PLEK, FCGR2A, FCGR1A, HCK, CYBB, CD86, ITGB2), which were selected from FCGR2A co-expressed DEGs. * p < 0.05; *** p < 0.001.

A total of 589 DEGs were identified from the TCGA-LIHC dataset under the criteria of |log_2_ fold change| > 1.5 and adjusted p-value < 0.05, including 430 upregulated and 159 downregulated genes. A volcano plot was generated to visualize the global transcriptional alterations (**Figure 5A**), and a heatmap illustrating the top 10 upregulated and downregulated DEGs, along with FCGR2A, was constructed using the pheatmap R package (**Figure 5B**).

To refine the genes closely associated with FCGR2A, we intersected the DEGs from TCGA-LIHC with the gene set obtained from the combined GEO datasets, yielding 253 overlapping DEGs (**Supplementary Table S9**). Pearson correlation analysis was subsequently performed between FCGR2A and these overlapping DEGs. The top 20 genes with the highest absolute correlation coefficients were defined as FCGR2A co-expressed genes, including CD86, LAIR1, LILRB4, ALOX5AP, CYBB, SYK, ITGB2, FCGR1A, HCK, CD53, VSIG4, FPR1, PLAUR, PTPRO, MNDA, DOCK2, IL2RA, BCAT1, CCR1, and PLEK. Correlation heatmaps in both the TCGA (**Figure 5C**) and combined GEO datasets (**Figure 5D**) demonstrated consistent, robust positive correlations (r > 0), supporting the reliability of these co-expressed relationships.

We further performed expression comparison analysis of the nine top-ranked hub genes between FCGR2A-high and FCGR2A-low groups. All genes—MNDA, CCR1, PLEK, FCGR2A, FCGR1A, HCK, CYBB, CD86, and ITGB2—were significantly upregulated in the high-expression group (p < 0.001; **Figure 5E**).

To further characterize the co-expression relationships at the protein level, we constructed a PPI network of the 20 co-expressed genes using the STRING database (**Supplementary Figure S5A**). Five centrality algorithms—Closeness (**Supplementary Figure S5B**), Degree (**Supplementary Figure S5C**), EPC (**Supplementary Figure S5D**), MCC (**Supplementary Figure S5E**), and MNC (**Supplementary Figure S5F**)—were applied via the CytoHubba plugin in Cytoscape to identify topological hub genes. A Venn diagram revealed nine genes commonly ranked among the top 10 by all algorithms (**Supplementary Figure S5G**), confirming ITGB2, CD86, CYBB, HCK, FCGR1A, FCGR2A, PLEK, CCR1, and MNDA as hub genes closely connected to FCGR2A in LIHC.

### Hub gene–based subtyping and immune microenvironment characterization

**Figure 6:**
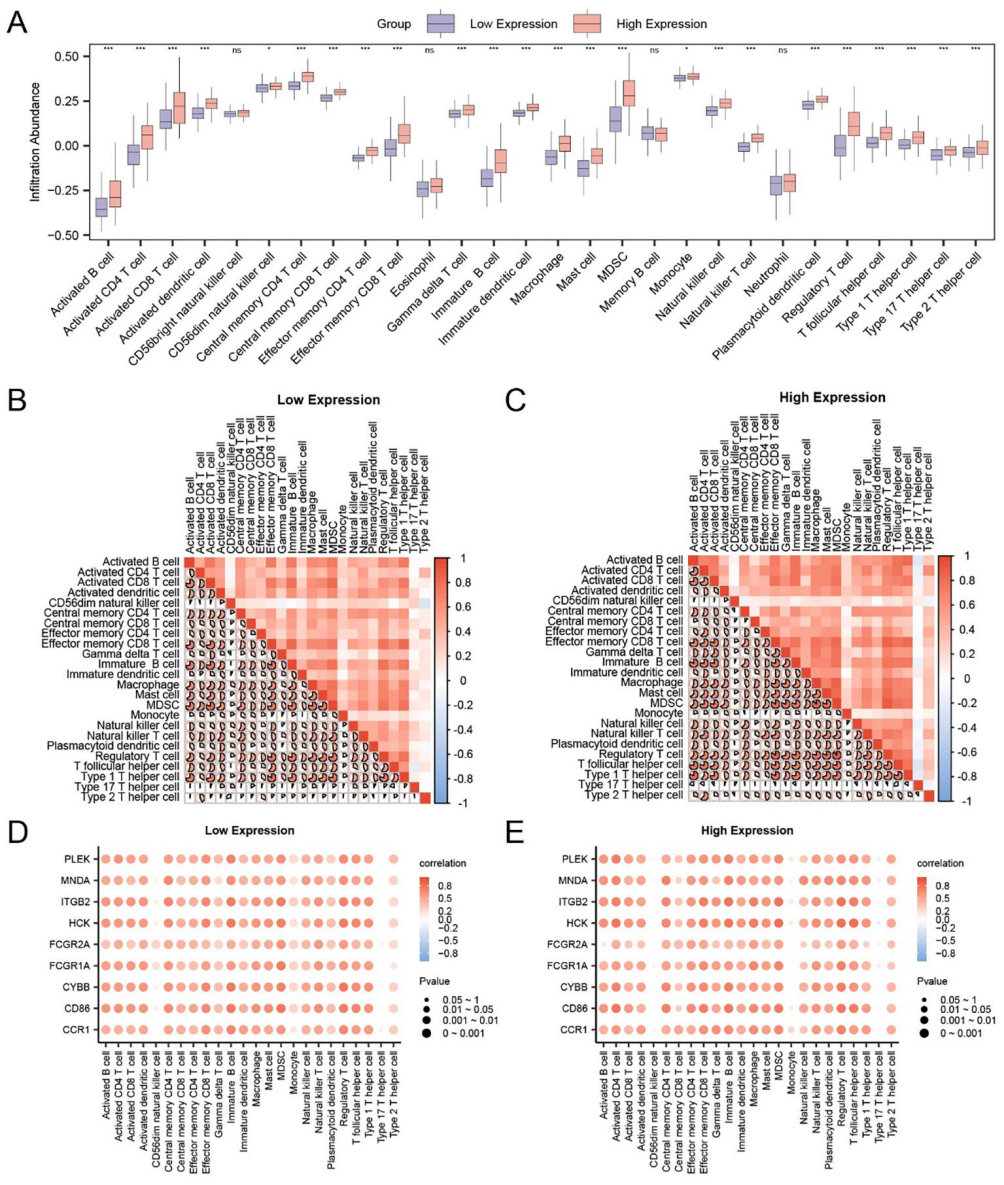
Correlation between hub genes and immune infiltration in HCC. **(A)** Comparison of immune cell infiltration levels between FCGR2A-high and -low expression groups using ssGSEA. **(B, C)** Heatmaps of immune cell correlations within FCGR2A low **(B)** and high **(C)** expression groups. **(D, E)** Bubble plots showing correlations between hub genes and immune cell infiltration scores in FCGR2A-low **(D)** and -high **(E)** groups. * p < 0.05; *** p < 0.001; ns, not significant.

To elucidate the immunological implications of FCGR2A and its co-expressed hub genes, we assessed immune cell infiltration in the TCGA-LIHC cohort using ssGSEA. A total of 28 immune cell types were profiled across the FCGR2A-high and -low groups. Notably, 24 subsets displayed significantly different infiltration levels (p < 0.05; **Figure 6A**), including activated CD4^+^/CD8^+^ T cells, γδ T cells, central/effector memory T cells, macrophages, dendritic cells, regulatory T cells, natural killer cells, and T helper subtypes (Th1/Th2/Th17), indicating a substantial shift in the immune landscape based on FCGR2A expression.

To evaluate internal coordination among immune subtypes, pairwise correlation heatmaps were generated within each expression group. In the low-expression group (**Figure 6B**), strong positive correlations were observed, most prominently between effector memory CD8^+^ T cells and Th1 cells (r = 0.823, p < 0.05). In the high-expression group (**Figure 6C**), a highly correlated immune network was also evident, with the strongest link detected between Tregs and MDSCs (r = 0.894, p < 0.05).

Finally, we evaluated the association between hub gene expression and immune infiltration using correlation bubble plots. In the low-expression group (**Figure 6D**), CYBB expression showed the strongest positive correlation with Treg abundance (r = 0.779, p < 0.05), while in the high-expression group (**Figure 6E**), HCK was most strongly correlated with T follicular helper cells (r = 0.817, p < 0.05). These findings suggest that FCGR2A and its immune-related co-expressed genes may participate in modulating distinct immune microenvironments depending on expression status.

### Hub gene–based subtyping reveals distinct immune profiles in HCC

**Figure 7:**
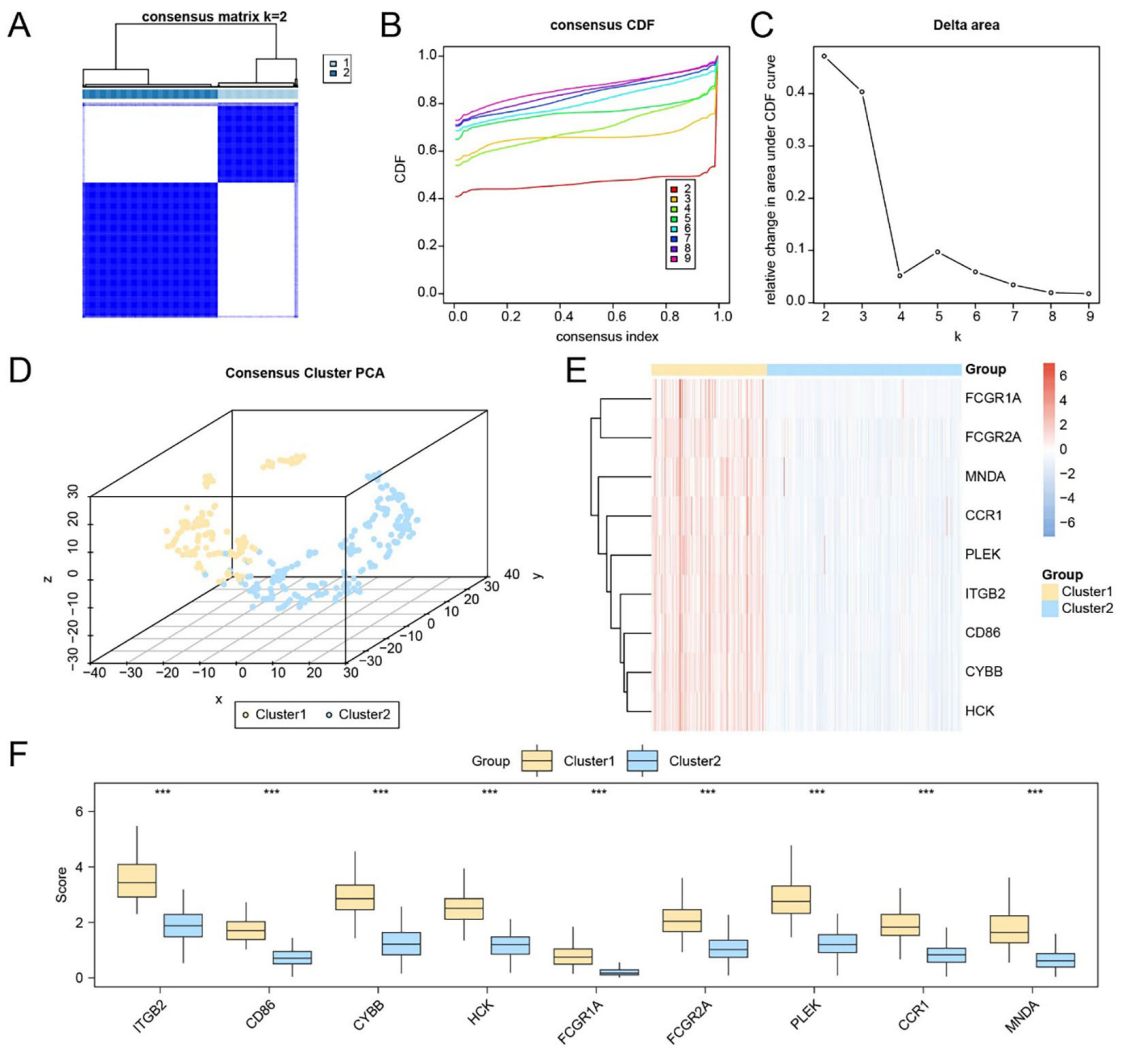
Identification of molecular subtypes based on the expression of hub genes. **(A)** Consensus matrix heatmap based on the expression of nine hub genes in the TCGA-LIHC dataset. **(B–C)** CDF curve **(B)** and delta area plot **(C)** used to determine the optimal number of clusters. **(D)** 3D t-SNE visualization of the two identified molecular subtypes. **(E)** Heatmap displaying the expression levels of hub genes across the two liver cancer subtypes. **(F)** Group comparison plot of hub gene expression between Subtype A (Cluster 1) and Subtype B (Cluster 2). *** p < 0.001.

To investigate potential molecular subtypes in HCC, consensus clustering was performed based on the expression profiles of nine hub genes (FCGR2A, ITGB2, CD86, CYBB, FCGR1A, HCK, MNDA, PLEK, and CCR1) using the Consensus ClusterPlus package. The optimal clustering partition divided the TCGA-LIHC cohort into two subtypes: Subtype A (Cluster 1, n = 137) and Subtype B (Cluster 2, n = 231), as supported by consensus heatmaps and CDF curves (**Figures 7A–C**). A 3D t-SNE projection further confirmed distinct separation between the two clusters (**Figure 7D**).

Expression heatmaps and statistical comparisons demonstrated that all nine hub genes were significantly differentially expressed between the two subtypes (**Figures 7E, F**; p < 0.001 for each gene). To characterize immunological differences between clusters, we applied the ssGSEA algorithm to quantify the infiltration levels of 28 immune cell types. Differential analysis revealed statistically significant differences (p < 0.05) in 27 immune cell subsets, including key components such as CD8^+^ T cells, regulatory T cells, dendritic cells, macrophages, NK cells, and memory T cells (**Supplementary Figure S6A**).

Correlation heatmaps showed extensive co-infiltration patterns within each subtype: in Subtype A, macrophages and MDSCs were most strongly correlated (r = 0.827), while in Subtype B, the strongest association was observed between Effector memory CD8^+^ T cells and Type 1 helper T cells (r = 0.721) (**Supplementary Figures S6B, C**). Additionally, gene–immune cell correlation bubble plots revealed that HCK expression was most strongly correlated with T follicular helper cells in Subtype A, whereas CD86 exhibited the strongest correlation with MDSCs in Subtype B (**Supplementary Figures S6D, E**).

### Subtype-specific immunotherapeutic implications and mutation landscape

**Figure 8:**
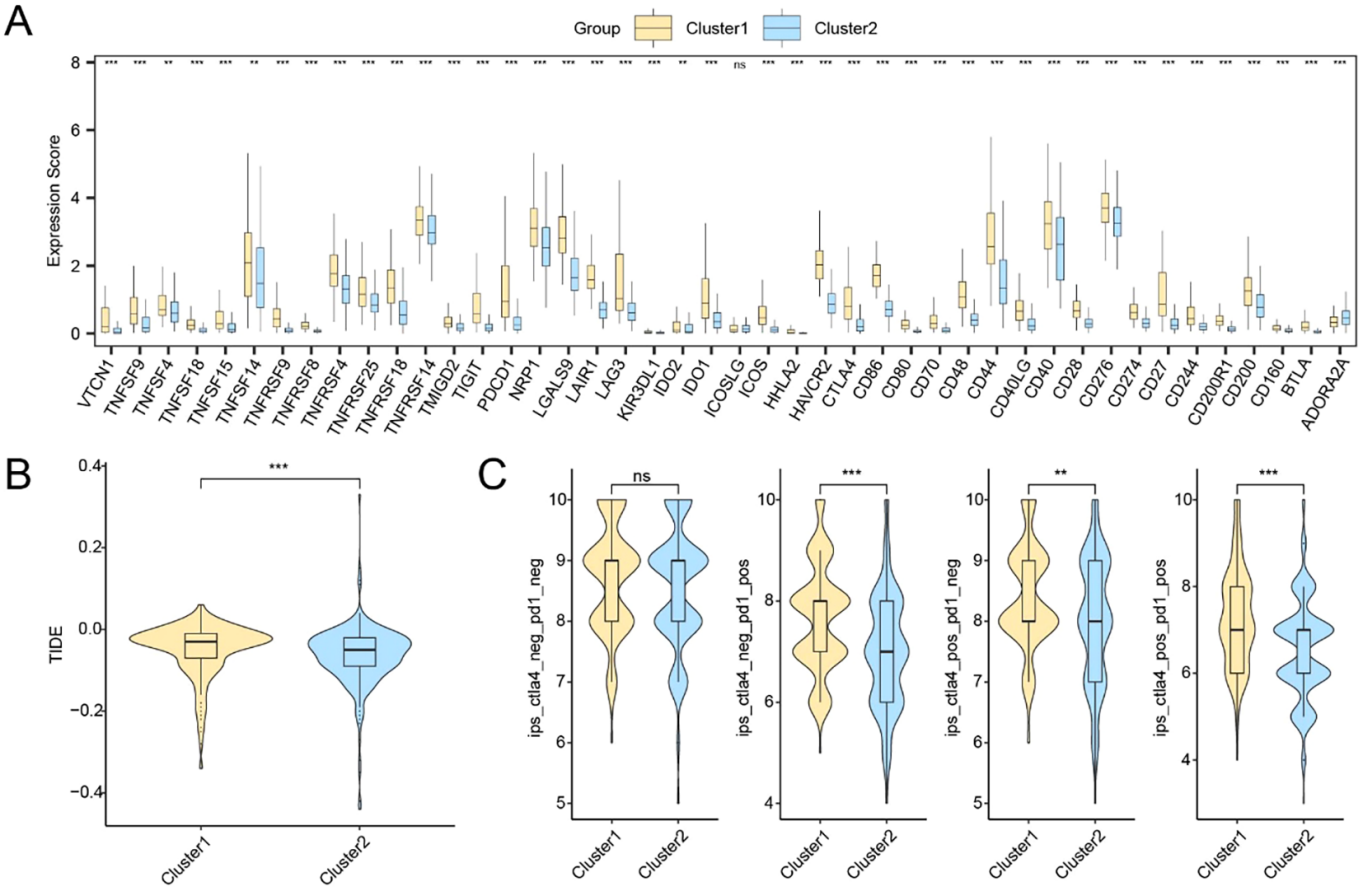
Immune checkpoint expression and predicted immunotherapy responses between HCC subtypes. **(A)** Expression profiles of immune checkpoint-related genes between Cluster1 and Cluster2. **(B)** TIDE scores in Cluster2. **(C)** IPS analysis under four immunotherapy scenarios. * p < 0.05; ** p < 0.01; *** p < 0.001; ns, not significant.

We next assessed the immunotherapy response potential and mutational characteristics associated with the two molecular subtypes. A total of 44 ICGs were evaluated, of which 41 showed statistically significant expression differences between Subtype A and Subtype B (**Supplementary Table S10**). Specifically, TNFSF4, TNFSF14, and IDO2 exhibited moderate significance (p < 0.01), while most others, including CD86, PDCD1, CTLA4, and TIGIT, showed highly significant differences (p < 0.001) (**Figure 8A**).

Furthermore, Subtype A exhibited a lower TIDE score, suggesting reduced immune evasion and potentially greater benefit from immunotherapy (p < 0.001; **Figure 8B**). IPS comparisons based on TCIA data also revealed statistically significant subtype differences in IPS components, indicating differential immune activation potential (**Figure 8C**).

We further analyzed somatic mutation data between High and Low FCGR2A expression groups. The most frequently mutated genes included TP53, CTNNB1, and TTN (**Supplementary Figure S7A**). Notably, CTNNB1 mutation frequency was significantly higher in the Low Expression group (p < 0.001; **Supplementary Figure S7D**). Functional enrichment of mutated genes revealed pathway preferences: WNT, RTK-RAS, and Hippo signaling were dominant in the Low Expression group (**Supplementary Figure S7B**), while RTK-RAS, TP53, and WNT pathways were enriched in the High Expression group (**Supplementary Figure S7C**).

Finally, genomic instability indicators including MSI and TMB were compared. MSI scores differed significantly between the two groups (p < 0.001; **Supplementary Figure S7E**), suggesting subtype-specific immunogenomic features potentially relevant to immune checkpoint blockade responsiveness.

## Discussion

HCC is a malignancy with pronounced immune heterogeneity. Despite the increasing clinical application of ICIs in recent years, their efficacy remains limited to a subset of patients, indicating the existence of complex immune evasion mechanisms and therapeutic resistance within the HCC immune microenvironment. This study presents a systematic research strategy that integrates IRDEG screening and functional interpretation, bridging data mining with functional validation. By integrating multiple transcriptomic datasets of HCC, we identified 21 IRDEGs closely associated with immune regulation and established a prognostic model centered on FCGR2A, achieving high predictive accuracy (AUC > 0.9). These findings offer theoretical support for biomarker discovery and individualized therapeutic strategies in HCC immunotherapy. Among the identified IRDEGs, several genes such as BIRC5, MAGEA1, and PRAME have been previously implicated in immune regulation across multiple solid tumors. For instance, Survivin encoded by BIRC5 not only modulates cell cycle progression but also contributes to immune escape by upregulating PD-L1 expression (45, 46);MAGEA1 and PRAME, as classical cancer-testis antigens, possess strong immunogenicity and have been recognized as effective targets for TCR-T cell therapies under specific HLA contexts (47, 48). These genes exhibit dual properties—oncogenicity and antigenicity—which may render them preferentially activated under immune-suppressive conditions, thereby promoting tumor progression and immune evasion simultaneously (49). Thus, the expression profile of IRDEGs not only holds prognostic value but may also reflect critical immune selective pressures shaping the tumor microenvironment.

Functional enrichment analysis revealed that IRDEGs are significantly enriched in cytokine-mediated signaling pathways, including classical inflammatory cascades such as JAK/STAT and NF-κB, and crosstalk with oncogenic pathways such as PI3K/AKT and RAS/MAPK (50). This signaling interplay suggests a potential mechanism by which immune and proliferative cues jointly shape the HCC phenotype. Based on consensus clustering, we further stratified HCC patients into two immune subtypes: an immune-inflamed phenotype enriched for immune response and chemotaxis signals, and an immune-excluded phenotype characterized by immunosuppressive pathways such as Wnt/β-catenin and TGF-β. This classification aligns with previously reported immune phenotypes in liver cancer and provides a conceptual framework for explaining heterogeneous responses to ICIs while informing combination treatment strategies (51, 52). Our FCGR2A-centered classification complements existing immune subtyping frameworks by providing a molecularly grounded, gene-specific perspective that links immune activation patterns with clinical outcomes, thereby enhancing its potential translational relevance. FCGR2A emerged as a central node in the PPI network, interacting with multiple immune-related proteins such as SYK, ITGB2, and FCGR3A (53). Previous studies have shown that FCGR2A, a low-affinity Fcγ receptor, plays a key role in ADCP mediated by macrophages and regulates myeloid cell polarization via Fc signaling pathways (54, 55). We validated the expression pattern and biological functions of FCGR2A through IHC and *in vitro* assays, demonstrating its significant upregulation in HCC tissues and its capacity to promote tumor cell proliferation, migration, and invasion. Given its upstream interaction with SYK and the established downstream activation of PI3K/AKT and NF-κB signaling, it is plausible that FCGR2A may drive tumor progression through these canonical immune-related pathways. This mechanistic axis represents a promising direction for further exploration to delineate how FCGR2A integrates immune recognition with oncogenic signaling in HCC. These findings suggest that FCGR2A may represent an adaptive response to immune pressure and function as a central mediator linking immune recognition, signal transduction, and tumor cell behavior.

From a translational perspective, FCGR2A has potential as both a diagnostic biomarker in immune pathology and a therapeutic target. Recent advances in antibody Fc engineering—such as enhancing FcγR binding affinity—have been employed to improve ADCC, and in this context, modulating FCGR2A expression or function may provide a novel entry point to boost ICI responsiveness in HCC (56). Furthermore, the prognostic model incorporating FCGR2A demonstrated excellent risk stratification capacity (AUC > 0.9), and could be integrated with traditional staging systems such as BCLC or TNM to form a dual-layer molecular-clinical predictive framework, thereby enhancing the precision of patient stratification and therapeutic decision-making (57). Despite the comprehensive mechanistic and experimental validation in this study, several limitations remain. First, all bioinformatic analyses relied on publicly available datasets and lack validation in large-scale, multi-center cohorts. Future work will aim to expand these analyses across additional international datasets and prospective clinical samples to further validate the robustness and generalizability of our findings. Second, the IHC validation in this study was based on a relatively modest number of patient samples, and the *in vitro* functional assays were performed using a single HCC cell line (SNU-878). These factors may limit the generalizability of our experimental findings. Third, the cell-type–specific expression patterns and signaling roles of FCGR2A (e.g., in tumor-associated macrophages vs. tumor cells) warrant further investigation using single-cell and spatial transcriptomic technologies. Integrating these multi-resolution datasets could clarify whether FCGR2A-driven immune modulation is predominantly immune-cell–intrinsic or tumor-cell–associated. Finally, while *in vitro* assays have preliminarily confirmed its functional role, causal relationships and immunotherapeutic implications require further validation in organoid or animal models.

In conclusion, this study systematically delineates the role of FCGR2A in HCC immune regulation and tumor progression through multi-omics analysis, network modeling, and biological validation. Our findings establish FCGR2A as a key immunoregulatory molecule and propose a closed-loop strategy for biomarker discovery—from data mining to functional confirmation. Collectively, this study integrates multi-omics and functional validation to define FCGR2A as a mechanistically interpretable biomarker linking immune activation with clinical outcomes, offering potential guidance for personalized immunotherapy and advancing precision oncology in HCC.

## Data Availability

The original contributions presented in the study are included in the article/**Supplementary Material**. Further inquiries can be directed to the corresponding authors.

## Supplementary material

Supplementary Table S1 List of immune-related genes (IRGs) obtained from GeneCards using the keyword "Immunotherapy", filtered by protein-coding status and relevance score >3.

Supplementary Table S2 Immune checkpoint genes (ICGs) used for subtype comparison.

Supplementary Table S3 Differentially expressed genes (DEGs) identified from TCGA-LIHC dataset using DESeq2.

Supplementary Table S4 Gene interaction candidates predicted by GeneMANIA using FCGR2A as the seed gene.

Supplementary Table S5 mRNA–TF regulatory network pairs derived from ChIPBase.

Supplementary Table S6 mRNA–miRNA regulatory network.

Supplementary Table S7 mRNA–RBP interaction pairs based on StarBase predictions.

Supplementary Table S8 Predicted mRNA–drug interactions from the Comparative Toxicogenomics Database (CTD).

Supplementary Table S9 Overlapping DEGs shared between TCGA and GEO datasets.

Supplementary Table S10 Expression matrix of immune checkpoint genes (ICGs) for 368 TCGA-LIHC samples.
