# Supplementary material for: FCGR2A defines prognostic immune subtypes and drives tumor progression in hepatocellular carcinoma
Source: Front Immunol. 2025 Oct 24;16:1641420. doi: 10.3389/fimmu.2025.1641420 (PMC12592142; doi:10.3389/fimmu.2025.1641420)
Supplement: Supplementary file 2 [file DataSheet1.docx]

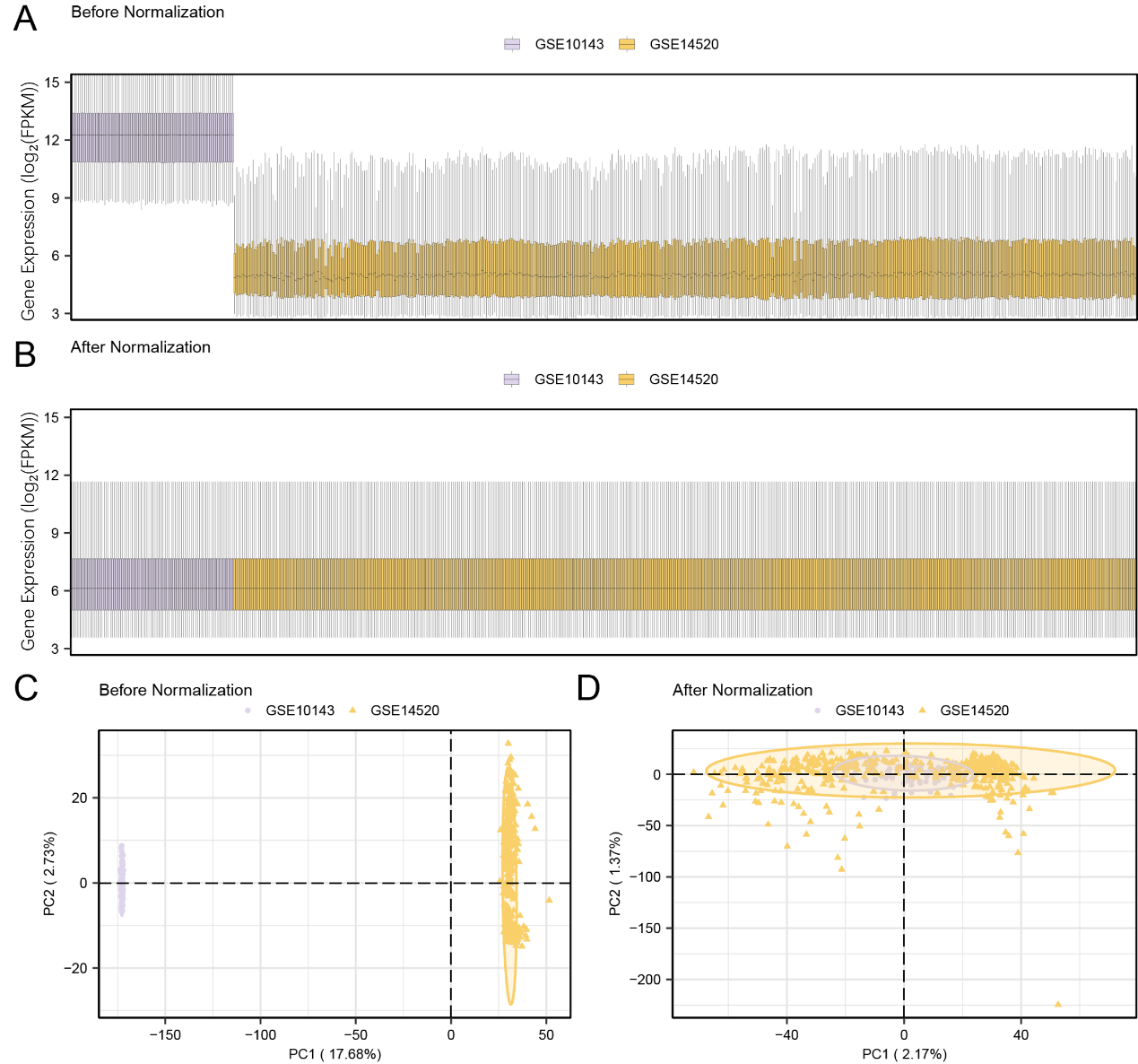


**Fig. S1. Batch effect removal and normalization of GSE10143 and GSE14520 Datasets**

(A–B) Boxplots showing expression distributions before (A) and after (B) batch effect correction of GSE10143 and GSE14520 datasets.
(C–D) Principal component analysis (PCA) before (C) and after (D) batch effect removal demonstrated successful integration of two datasets.


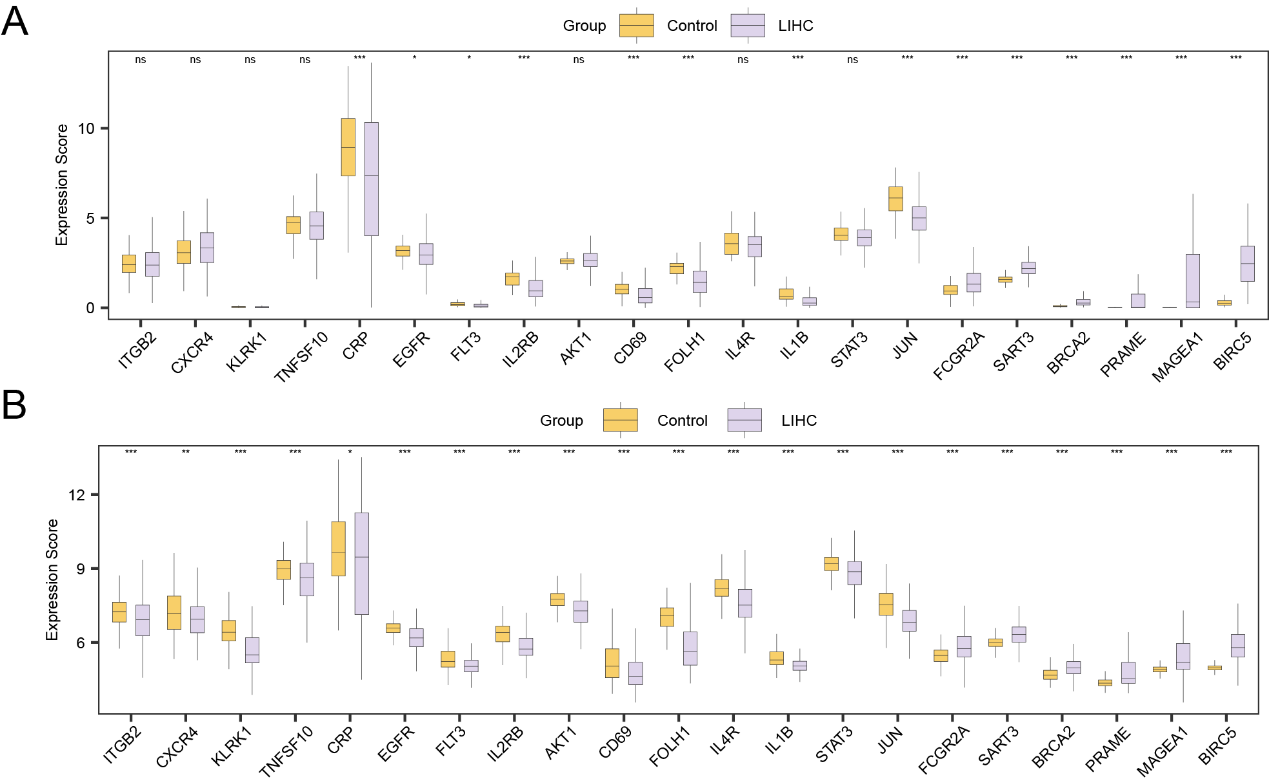


**Fig.S2. Expression validation of IRDEGs in LIHC.**(A) Heatmap of 21 immune-related differentially expressed genes (IRDEGs) in the TCGA-LIHC dataset.
(B) Heatmap of the same IRDEGs in the combined GEO datasets.
These results confirmed consistent overexpression of key IRDEGs across independent cohorts.


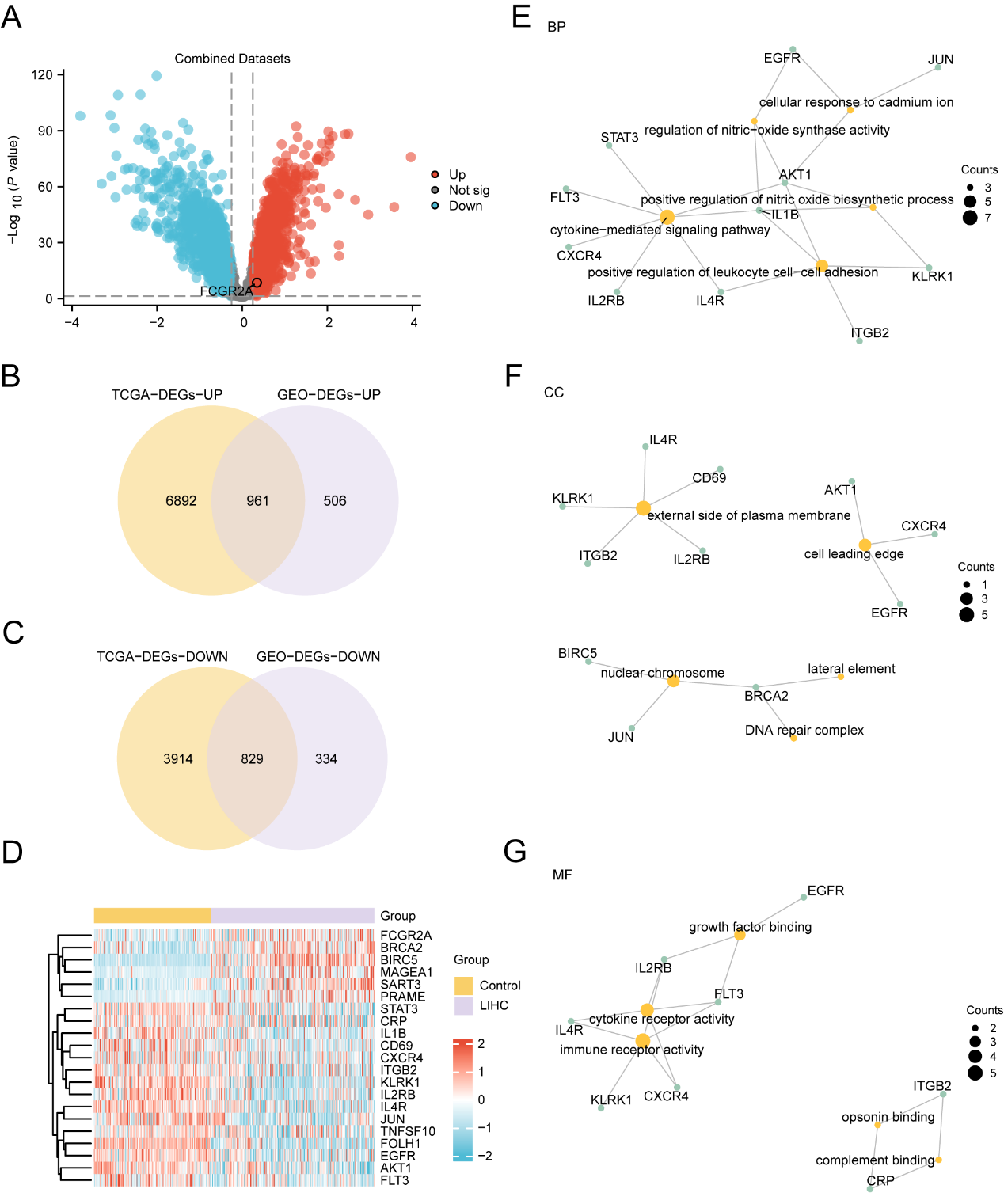


**Figure S3. Identification and functional enrichment of intersected IRDEGs.**

**(A)** Volcano plot of DEGs in the combined GEO datasets. FCGR2A is highlighted. Genes with |log₂FC| > 1.5 and *adj. p* < 0.05 were considered significant.

**(B–C)** Venn diagrams showing overlapping upregulated (B) and downregulated (C) genes between TCGA and GEO cohorts, used to identify robust DEGs.

**(D)** Heatmap showing the expression patterns of 21 IRDEGs in the GEO datasets. These genes were obtained by intersecting DEGs with immune-related genes (IRGs), and their consistent dysregulation in both datasets supports their immune relevance.

**(E–G)** GO enrichment network of the 21 IRDEGs:
**(E)** Biological Process (BP),
**(F)** Cellular Component (CC),
**(G)** Molecular Function (MF).


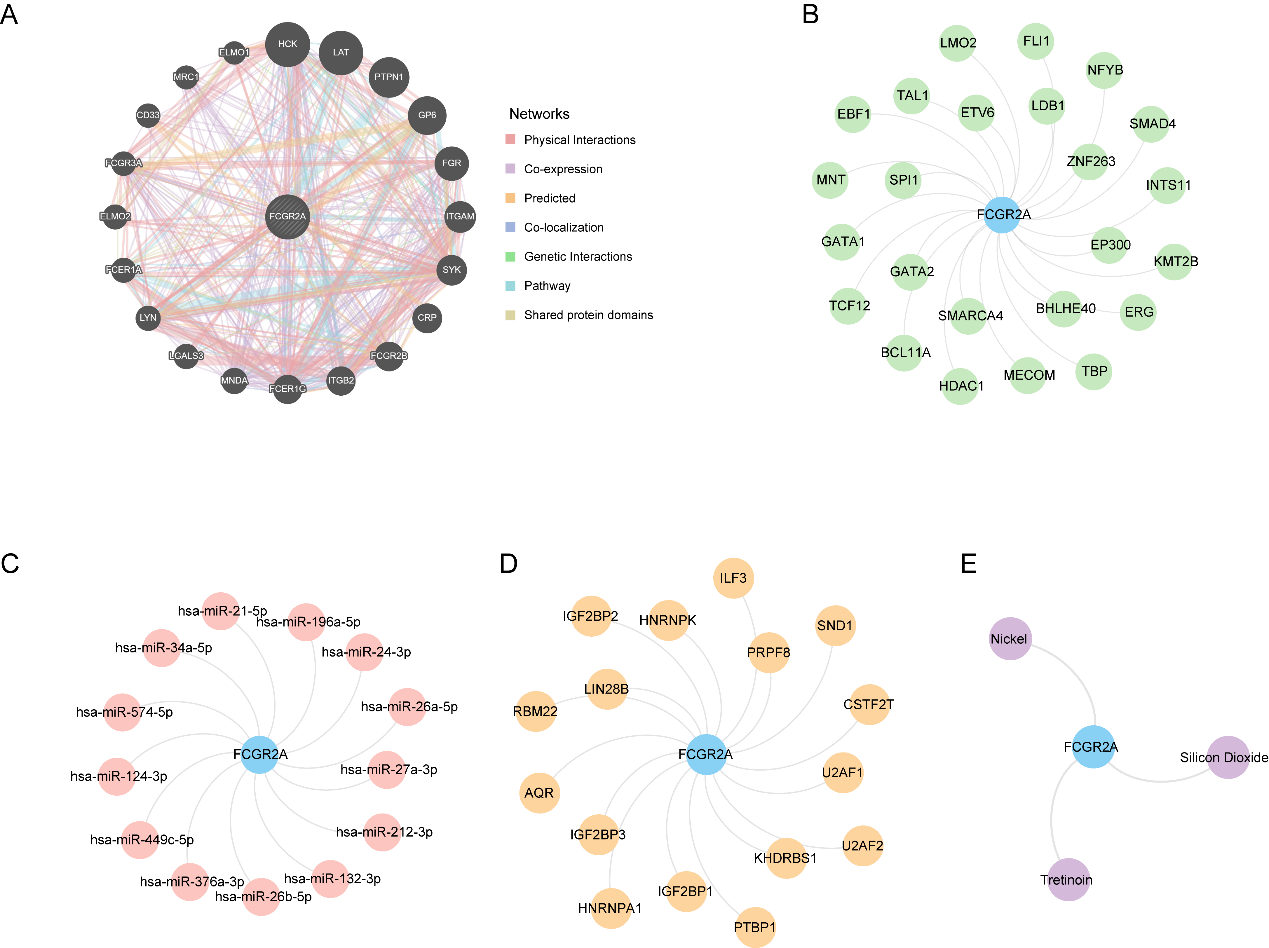


**Figure S4. Regulatory network and interacting partners of FCGR2A.**

(A) Protein–protein interaction (PPI) network of FCGR2A constructed using GeneMANIA, demonstrating multiple predicted and validated interactions including physical interactions, co-expression, and pathway associations.
(B) Predicted transcription factors (TFs) potentially regulating FCGR2A expression.
(C) Predicted microRNAs (miRNAs) that may target FCGR2A.
(D) RNA-binding proteins (RBPs) predicted to interact with FCGR2A mRNA.
(E) Chemical compounds potentially interacting with FCGR2A, suggesting possible regulatory or pharmacological relevance.


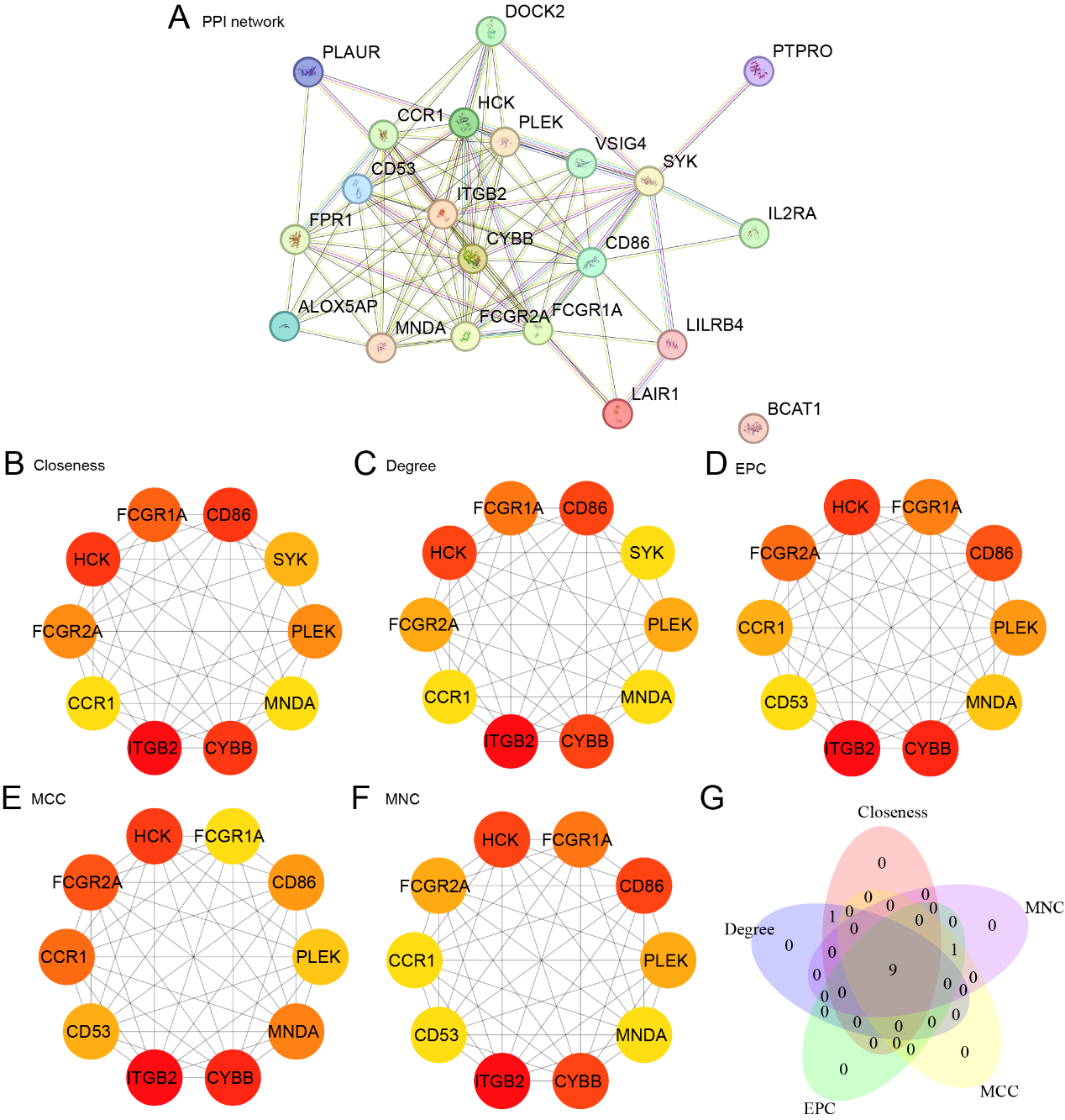


**Figure S5. Protein–protein interaction (PPI) network and hub gene identification.**

(A) PPI network constructed using STRING and visualized in Cytoscape based on FCGR2A-associated immune genes.
(B–F) Hub gene networks identified by five algorithms: Closeness, Degree, EPC, MCC, and MNC.
(G) Venn diagram showing overlap of top-ranked hub genes across five centrality methods.


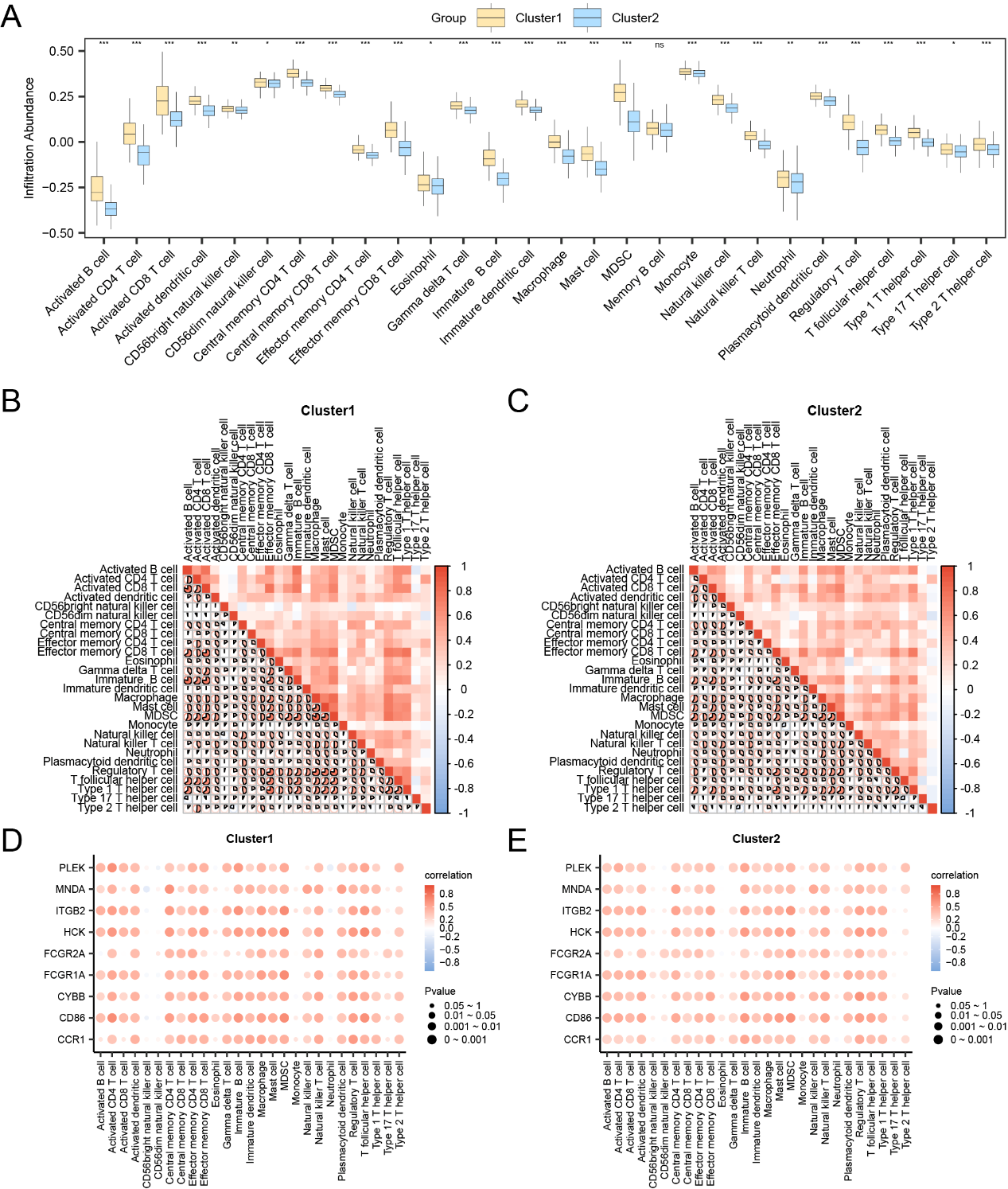


**Fig S6. Comparison of immune cell infiltration patterns between Cluster 1 and Cluster 2.**

(A) Boxplots showing the relative infiltration levels of 28 immune cell subtypes in Cluster 1 vs. Cluster 2.
(B–C) Heatmaps depicting correlation matrices of immune cell types within Cluster 1 (B) and Cluster 2 (C).
(D–E) Dot plots illustrating the correlation between the 10 hub genes and immune cell infiltration in Cluster 1 (D) and Cluster 2 (E).
Significant correlations (Pearson, *p* < 0.05) are marked by dot size, and color reflects the correlation coefficient.


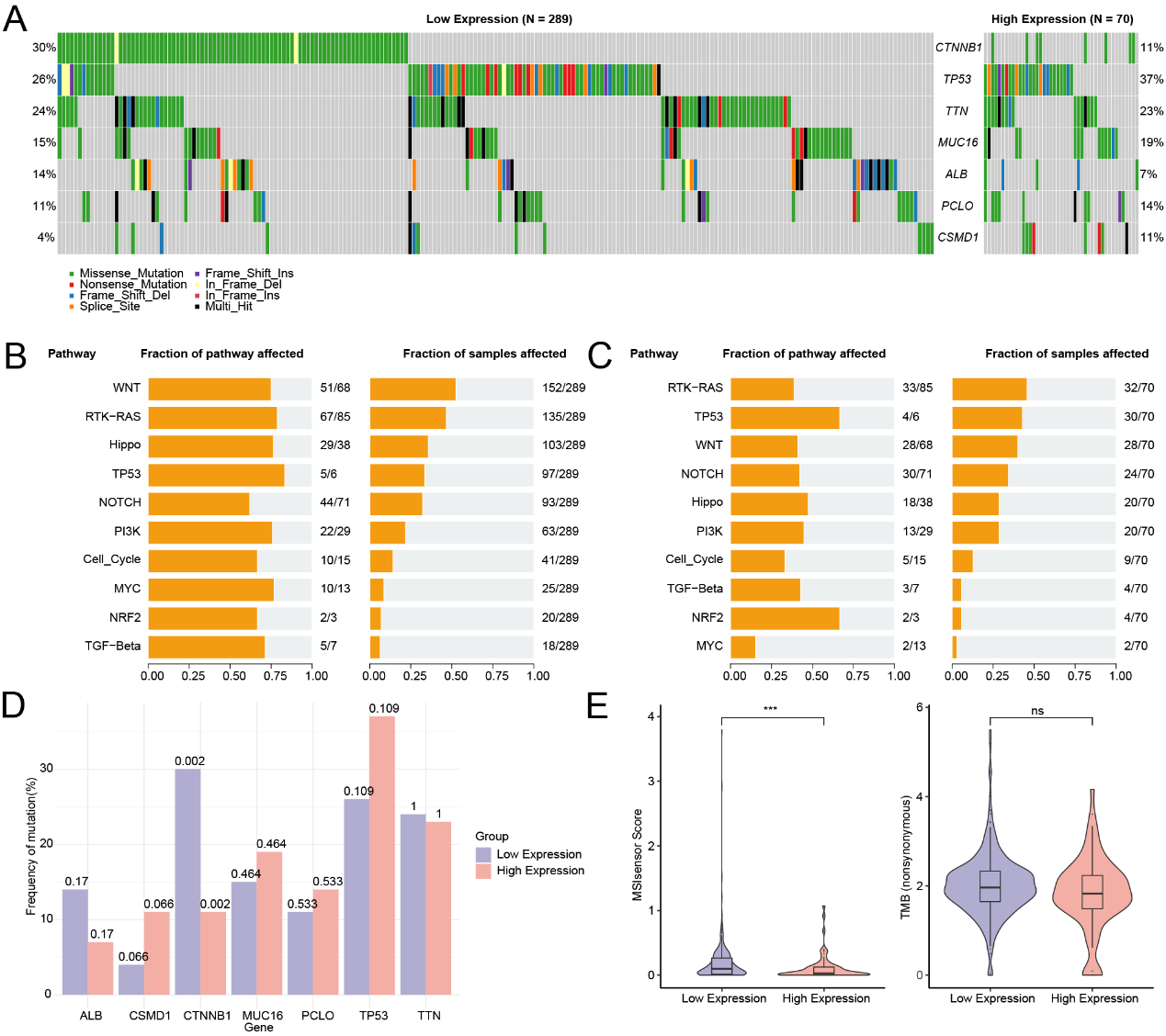


**Fig.S7. Genomic Alterations, MSI and TMB Analysis**

(A) Oncoplot showing the mutation landscape of LIHC samples in the High and Low Expression groups.
(B–C) Functional pathway enrichment of somatic mutations in the Low Expression group (B) and High Expression group (C), respectively.
(D) Bar chart comparing mutation frequencies of key genes between High and Low Expression groups.
(E) Group comparison of MSI scores and TMB scores between the High and Low Expression groups in the TCGA-LIHC dataset.


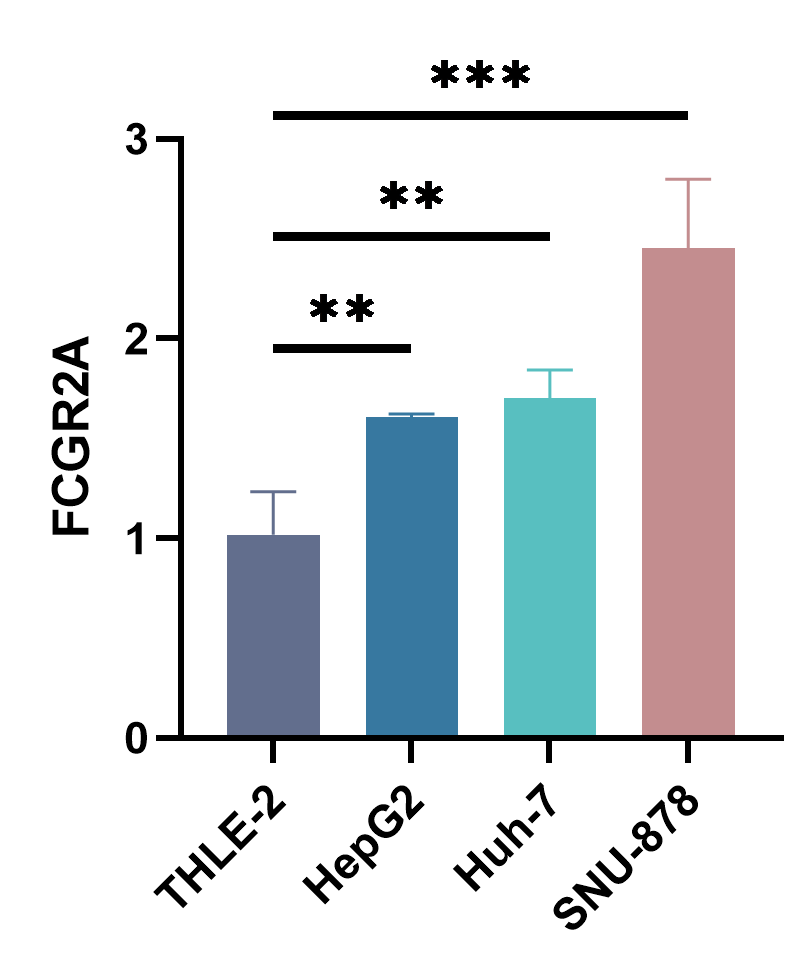


**Fig.S8. Baseline FCGR2A protein expression in normal hepatocytes and HCC cell lines.**
