# Supplementary figures and images for: FCGR2A defines prognostic immune subtypes and drives tumor progression in hepatocellular carcinoma
Source: Front Immunol. 2025 Oct 24;16:1641420. doi: 10.3389/fimmu.2025.1641420 (PMC12592142; doi:10.3389/fimmu.2025.1641420)

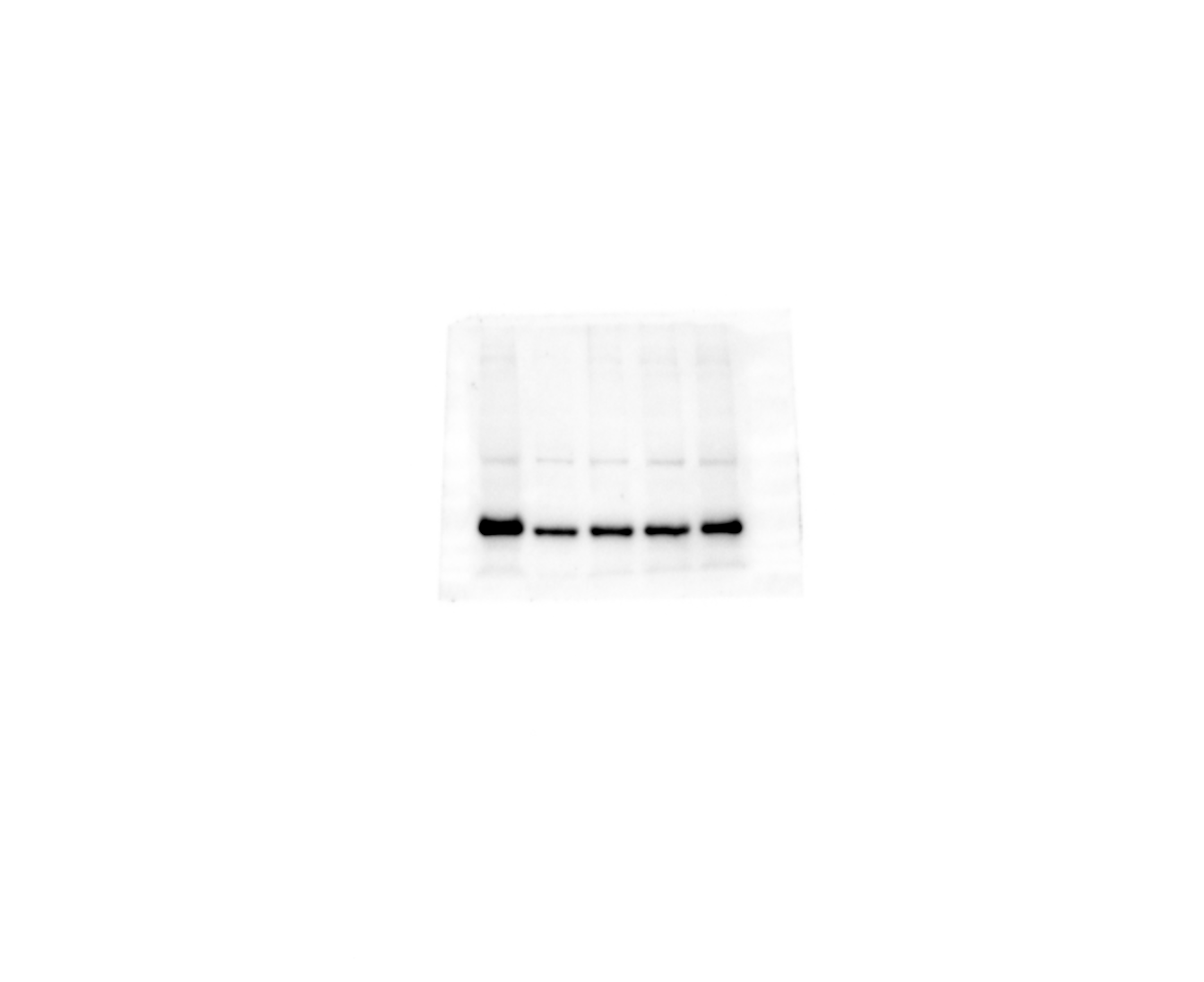

Supplement: Supplementary file 3 [file Image1.tif]

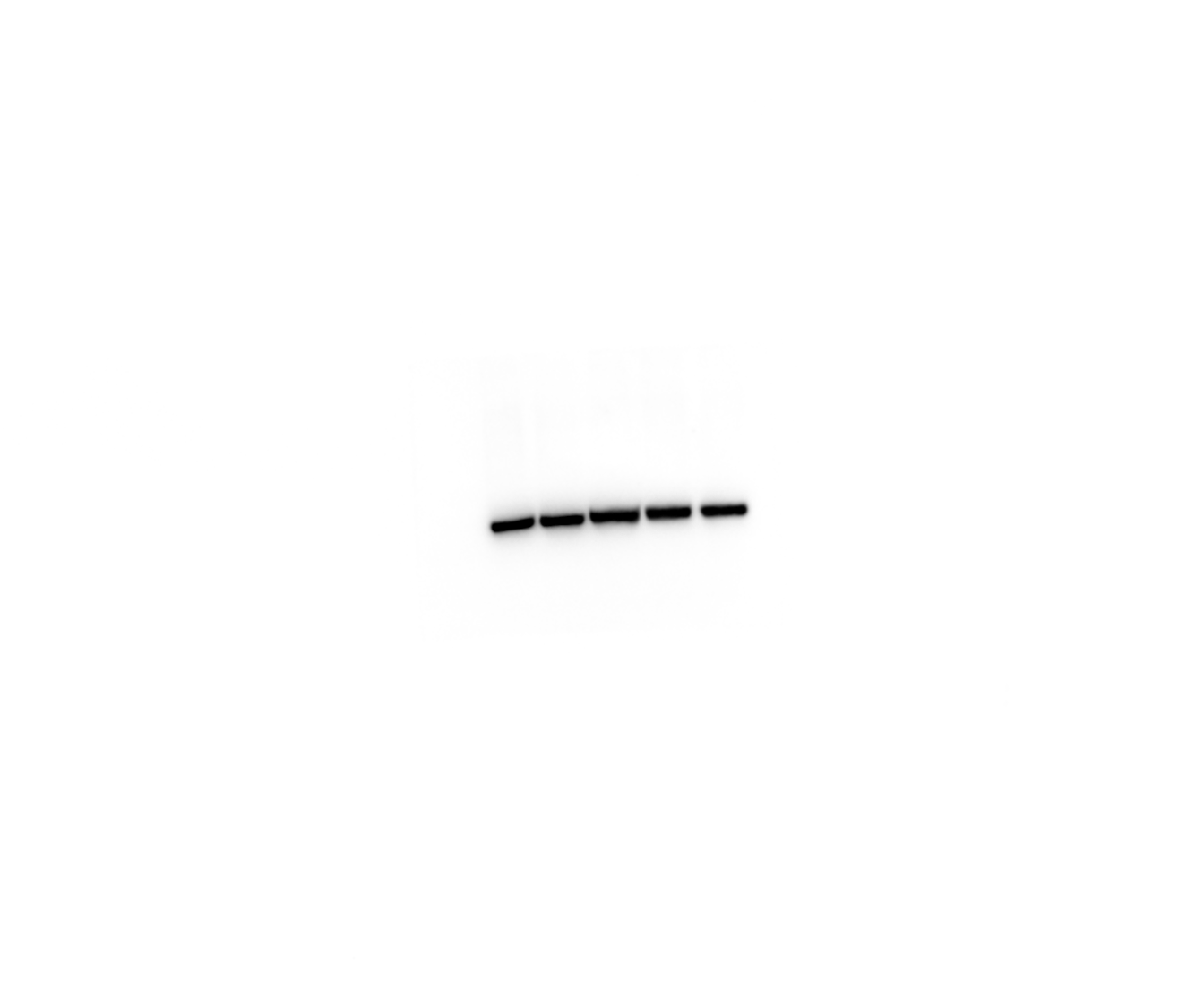

Supplement: Supplementary file 4 [file Image2.tif]
